# Supplementary material for: ERK1/2 inhibits Cullin 3/SPOP-mediated PrLZ ubiquitination and degradation to modulate prostate cancer progression
Source: Cell Death Differ. 2022 Feb 22;29(8):1611–24. doi: 10.1038/s41418-022-00951-y (PMC9345960; doi:10.1038/s41418-022-00951-y)
Supplement: Supplementary file 1 — Supplementary Figures [file 41418_2022_951_MOESM1_ESM.doc]

Supplementary Figures for

**ERK1/2 inhibits Cullin 3/SPOP-mediated PrLZ ubiquitination and degradation to modulate prostate cancer progression**

Yizeng Fan^1,3^, Tao Hou^1,3^, Weichao Dan^1,3^, Yasheng Zhu^2,3^, Bo Liu^1^, Yi Wei^1^, Zixi Wang^1^, Yang Gao^1^, Jin Zeng^1,*^, and Lei Li^1,*^

^1^ Department of Urology, The First Affiliated Hospital of Xi'an Jiaotong University, 710061, Xi'an, P. R. China.

^2^ Department of Urology, Shanghai Changhai Hospital, Second Military Medical University, 200433, Shanghai, P.R. China.

^3^ These authors contributed equally to this work.

^*^Correspondence and requests for materials should be addressed to J.Z. (zengjin1984@126.com); L.L. (E-mail: [lilydr@163.com)](mailto:lilydr@163.com)).

**This file includes:**

Supplementary Figure S1-S11

**Supplementary Figure S1**

**
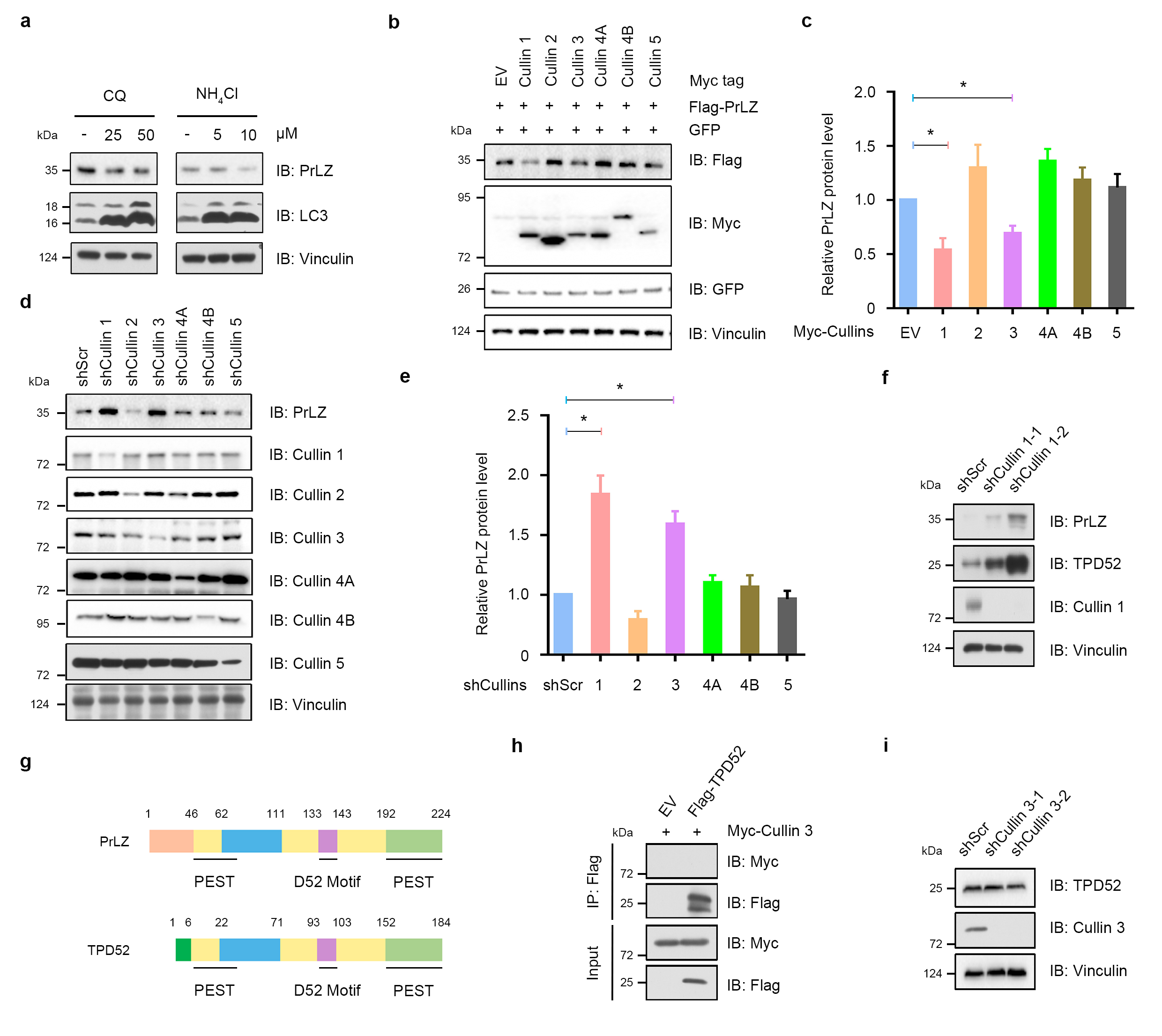
**

**Supplementary Figure S2**


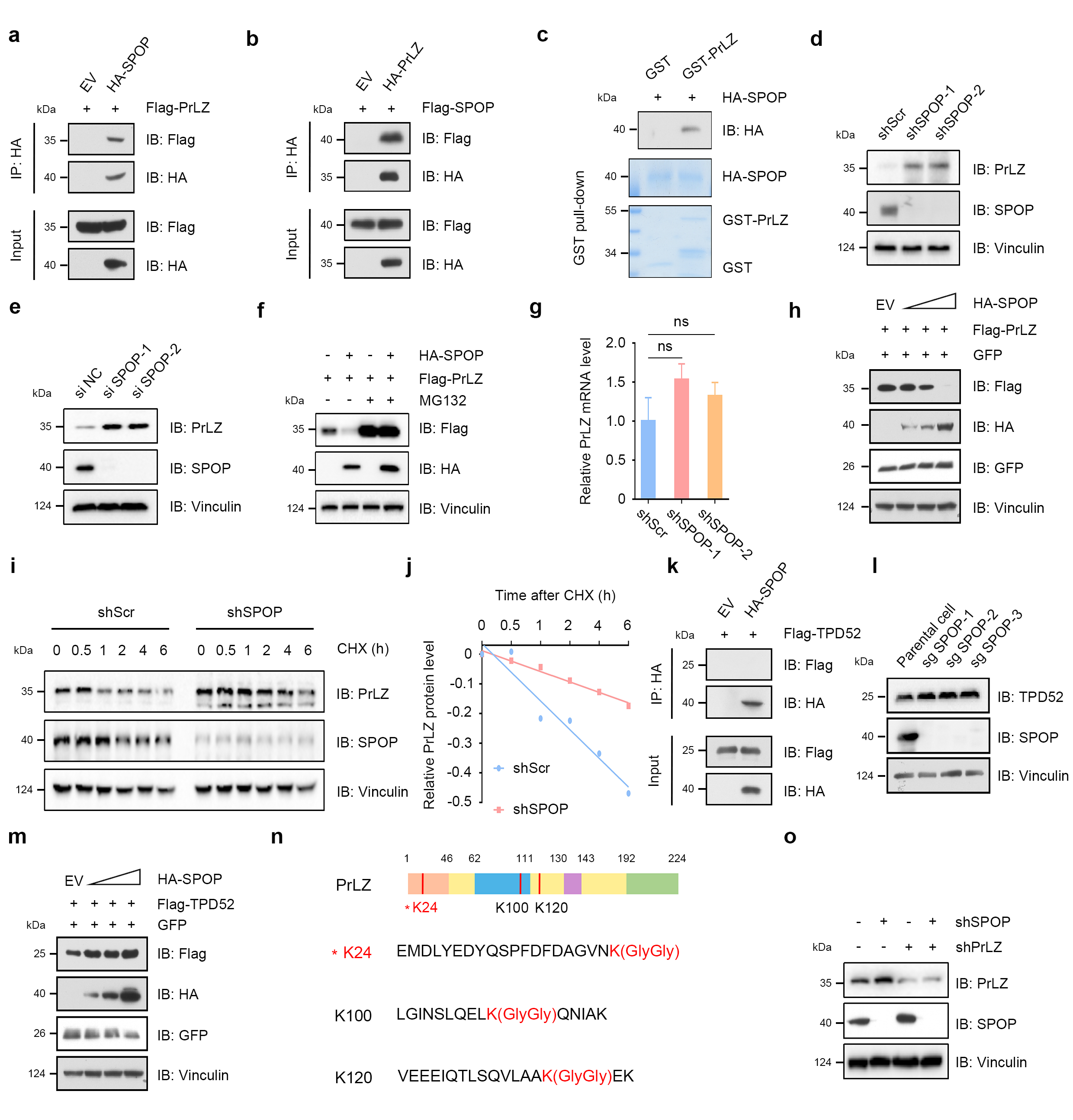


**Supplementary Figure S3**


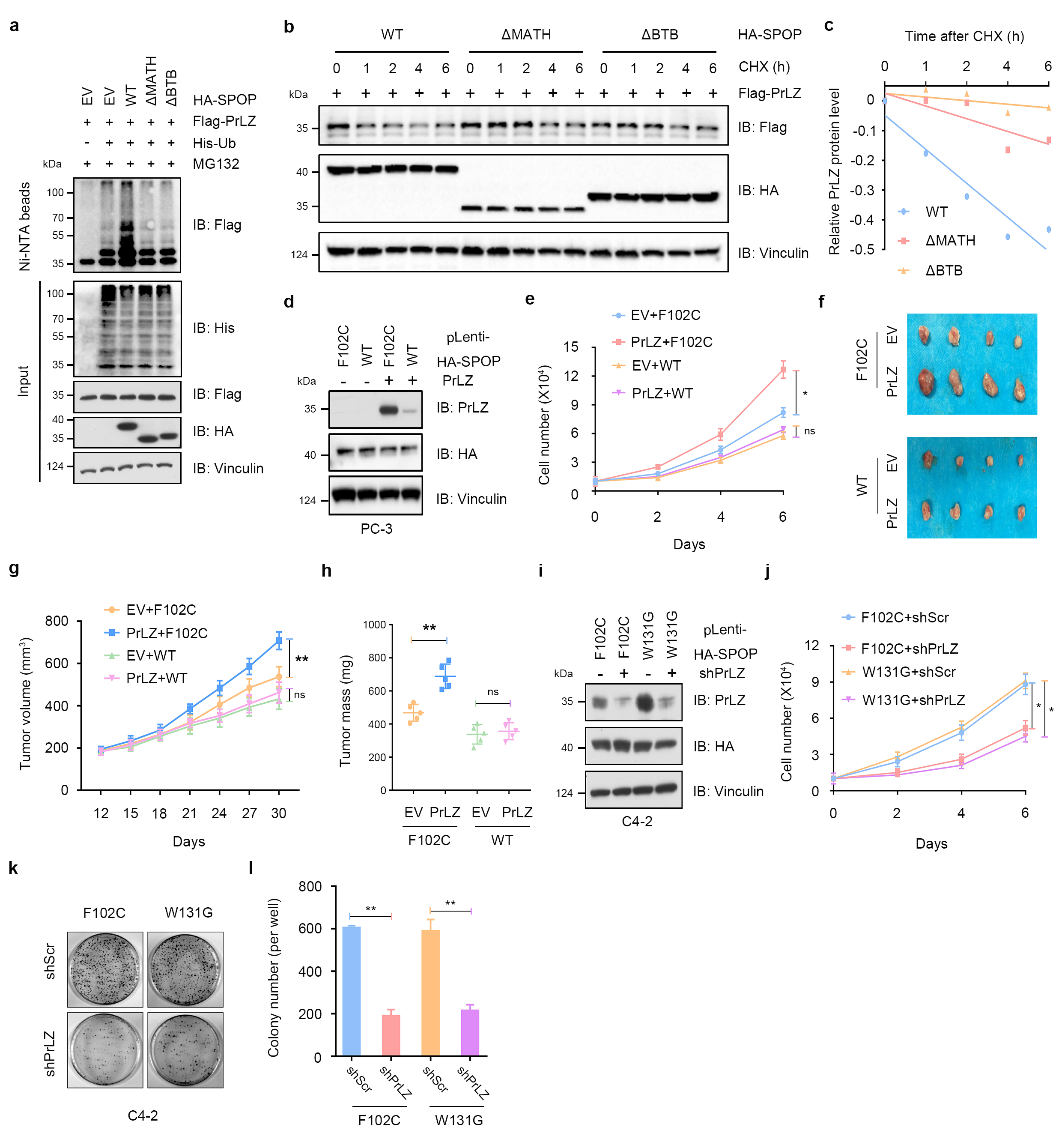


**Supplementary Figure S4**


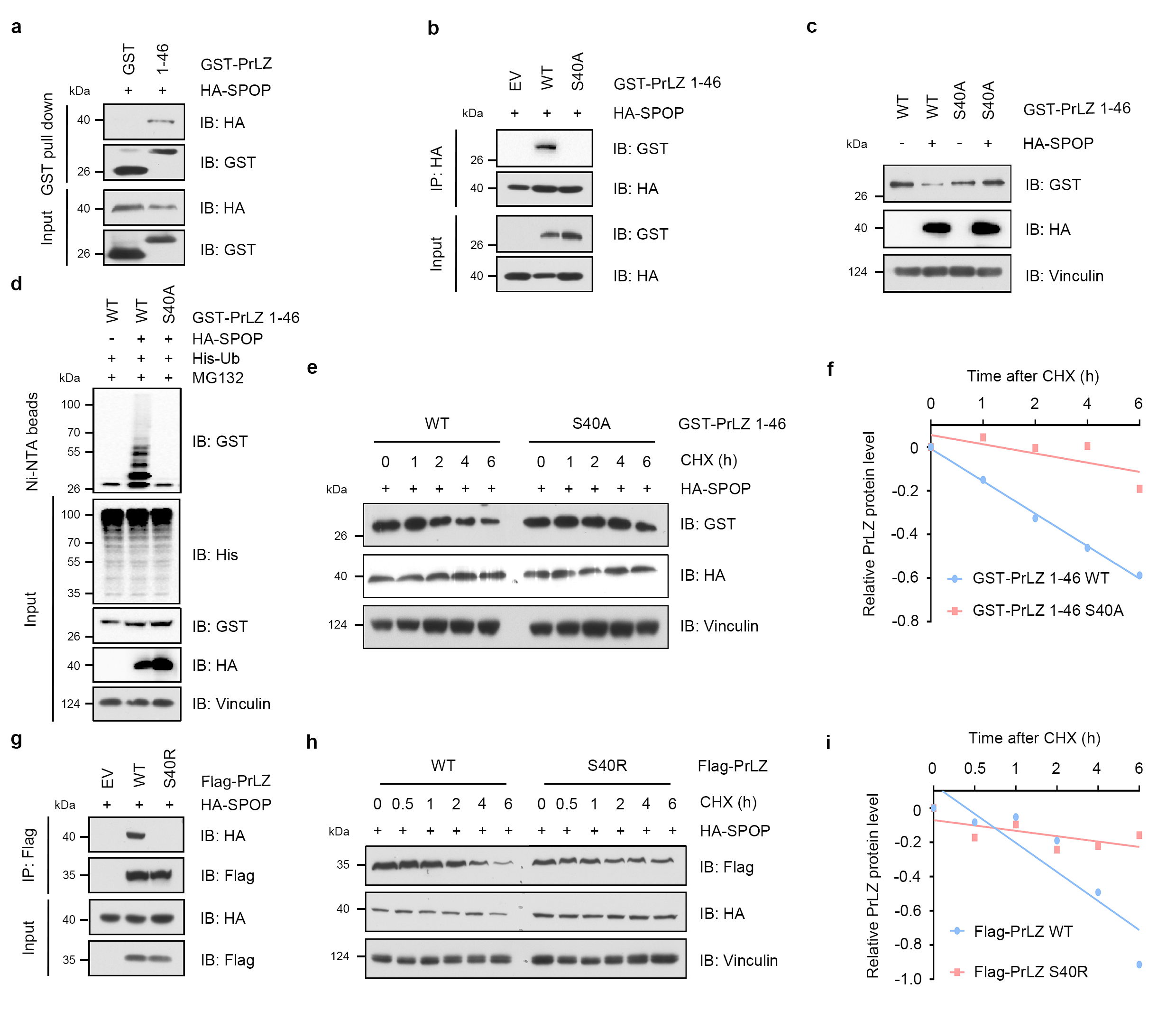


**Supplementary Figure S5**


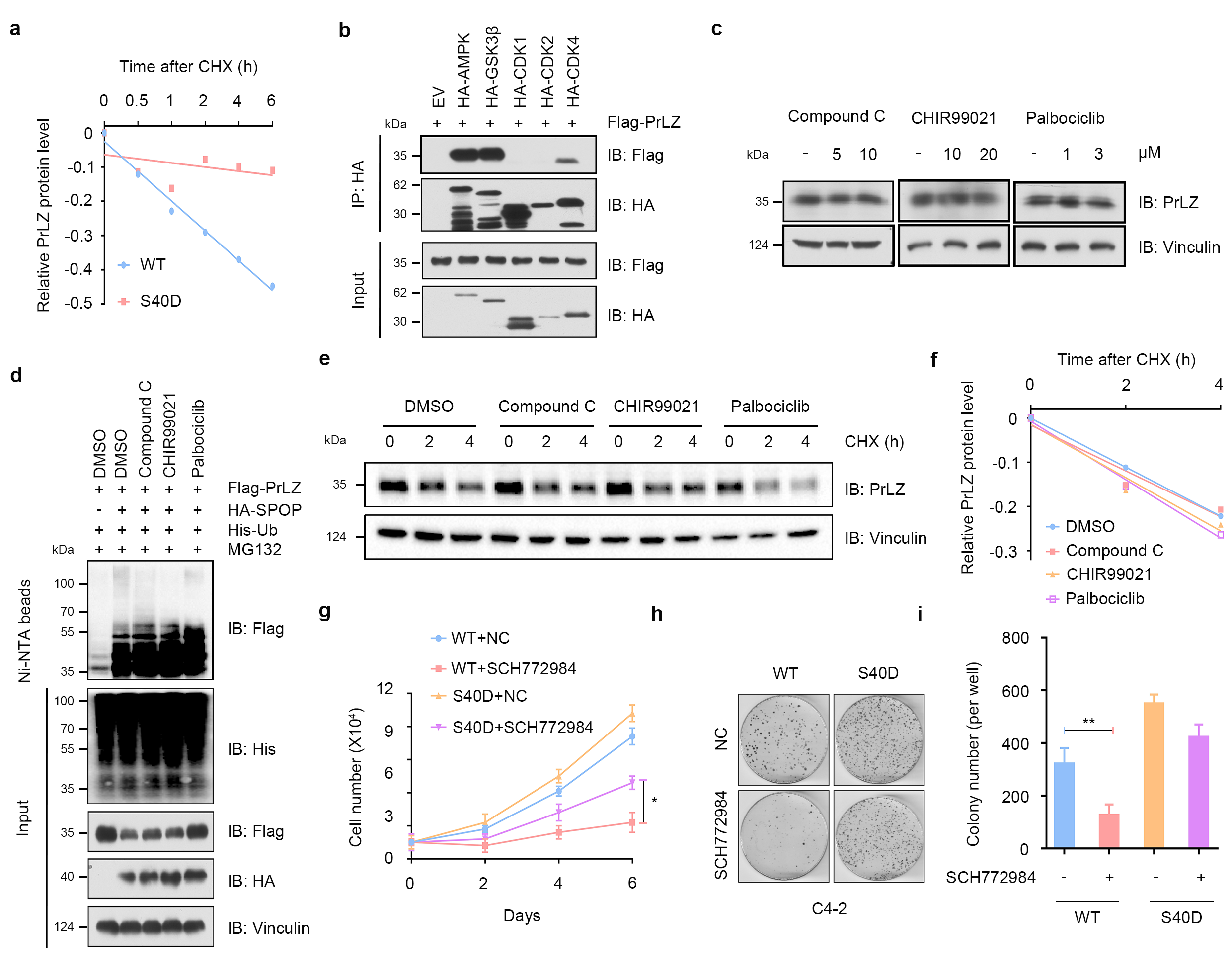


**Supplementary Figure S6**


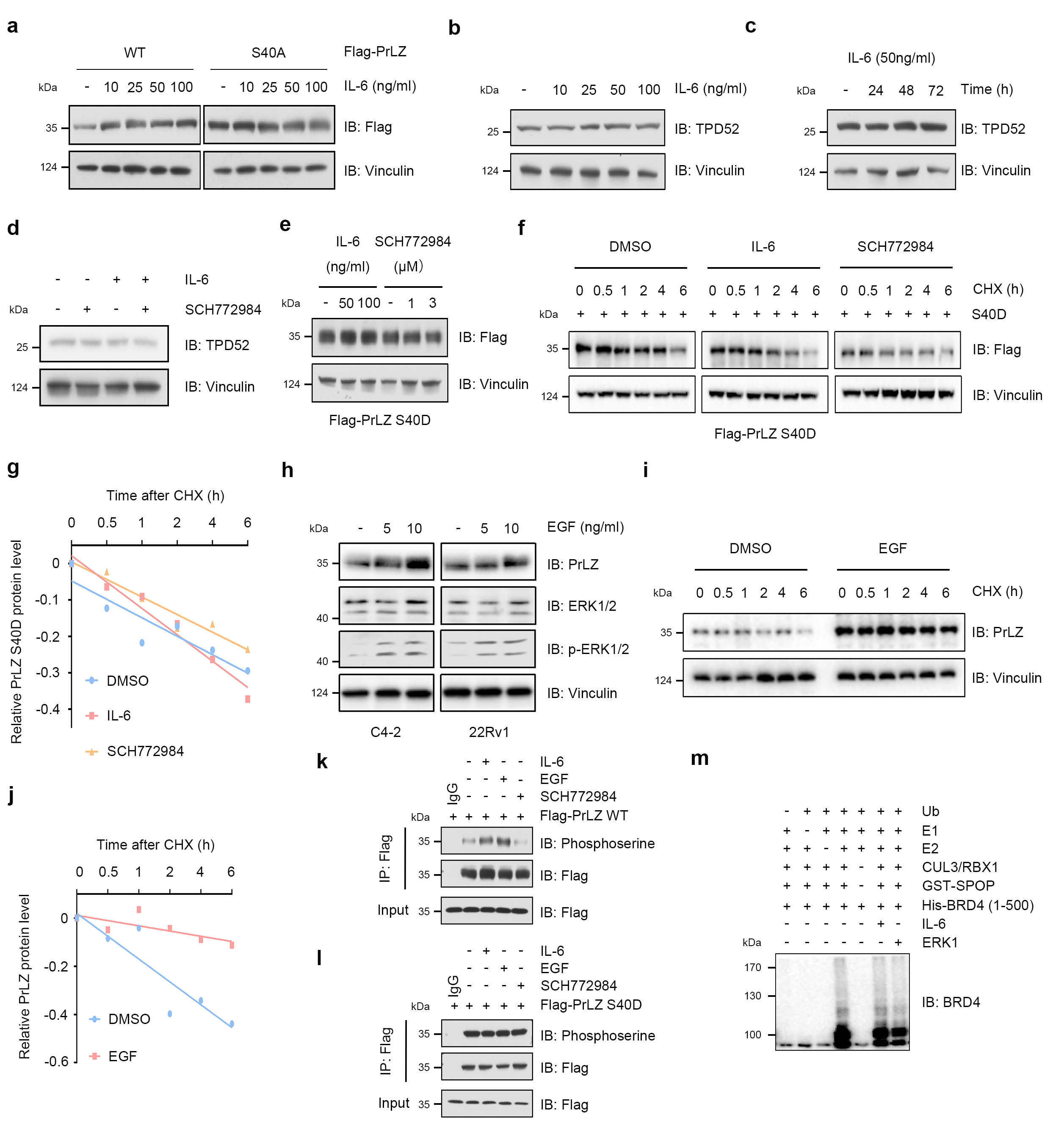


**Supplementary Figure S7**


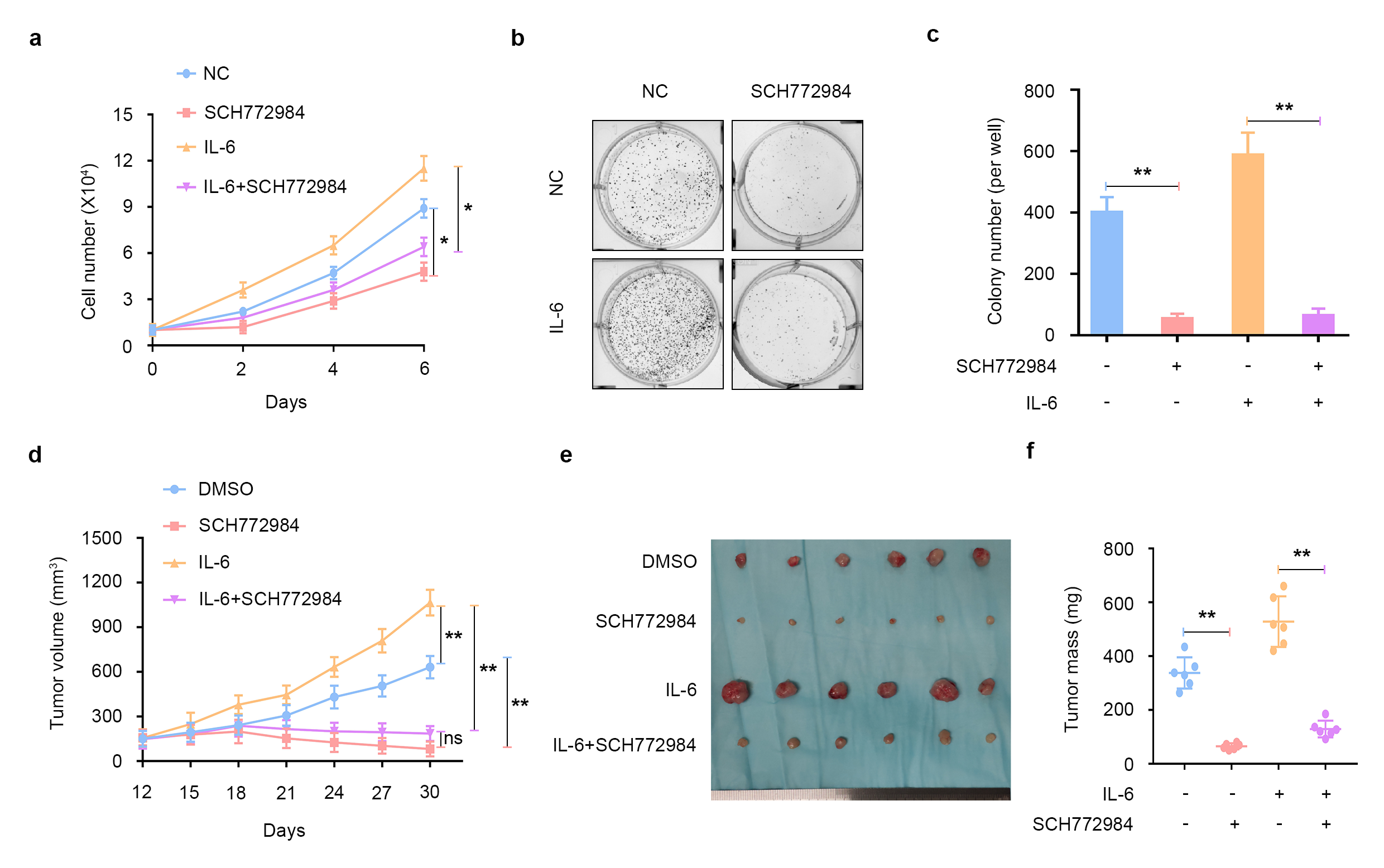


**Supplementary Figure S8**

**
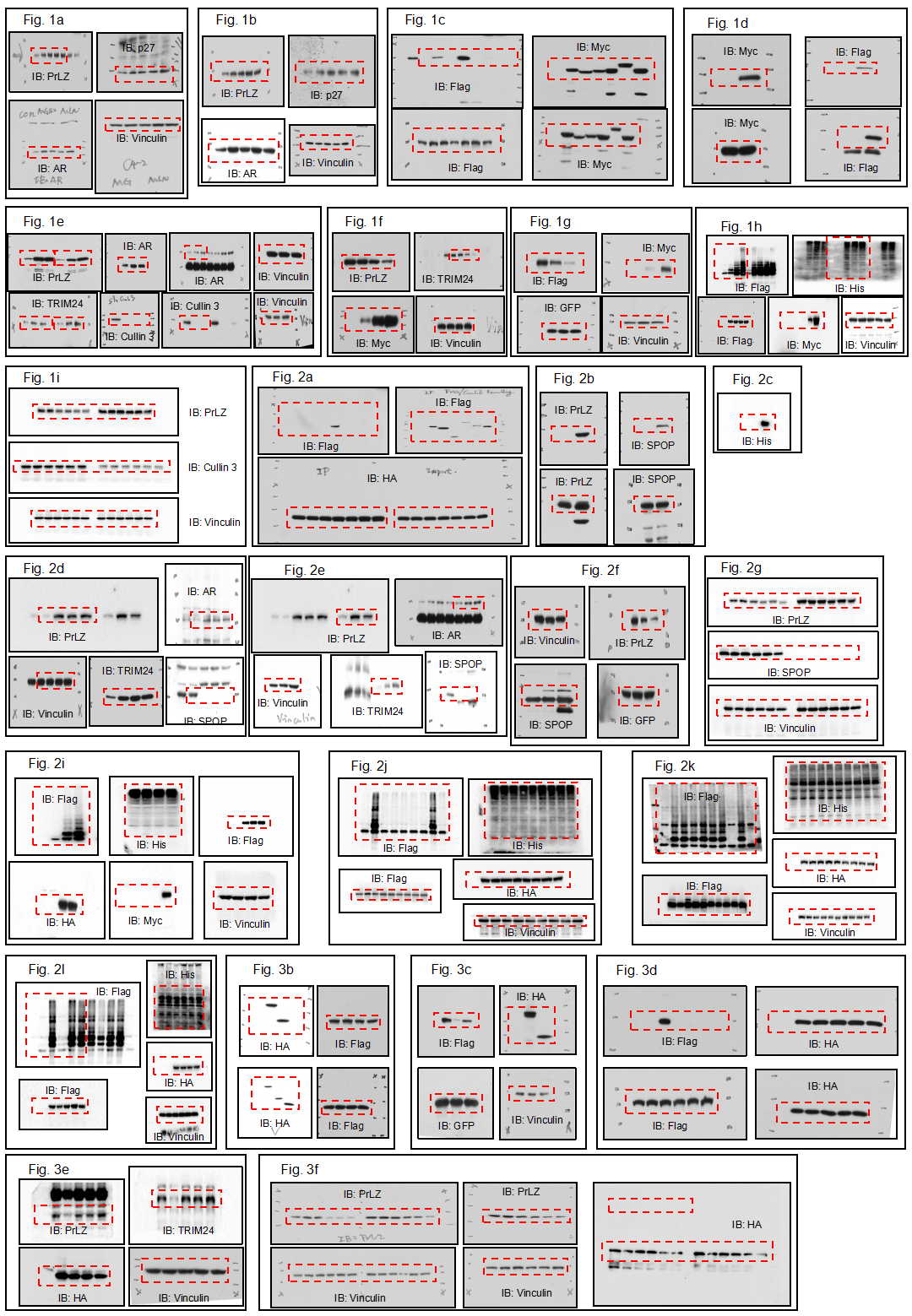
**

**Supplementary Figure S9**

**
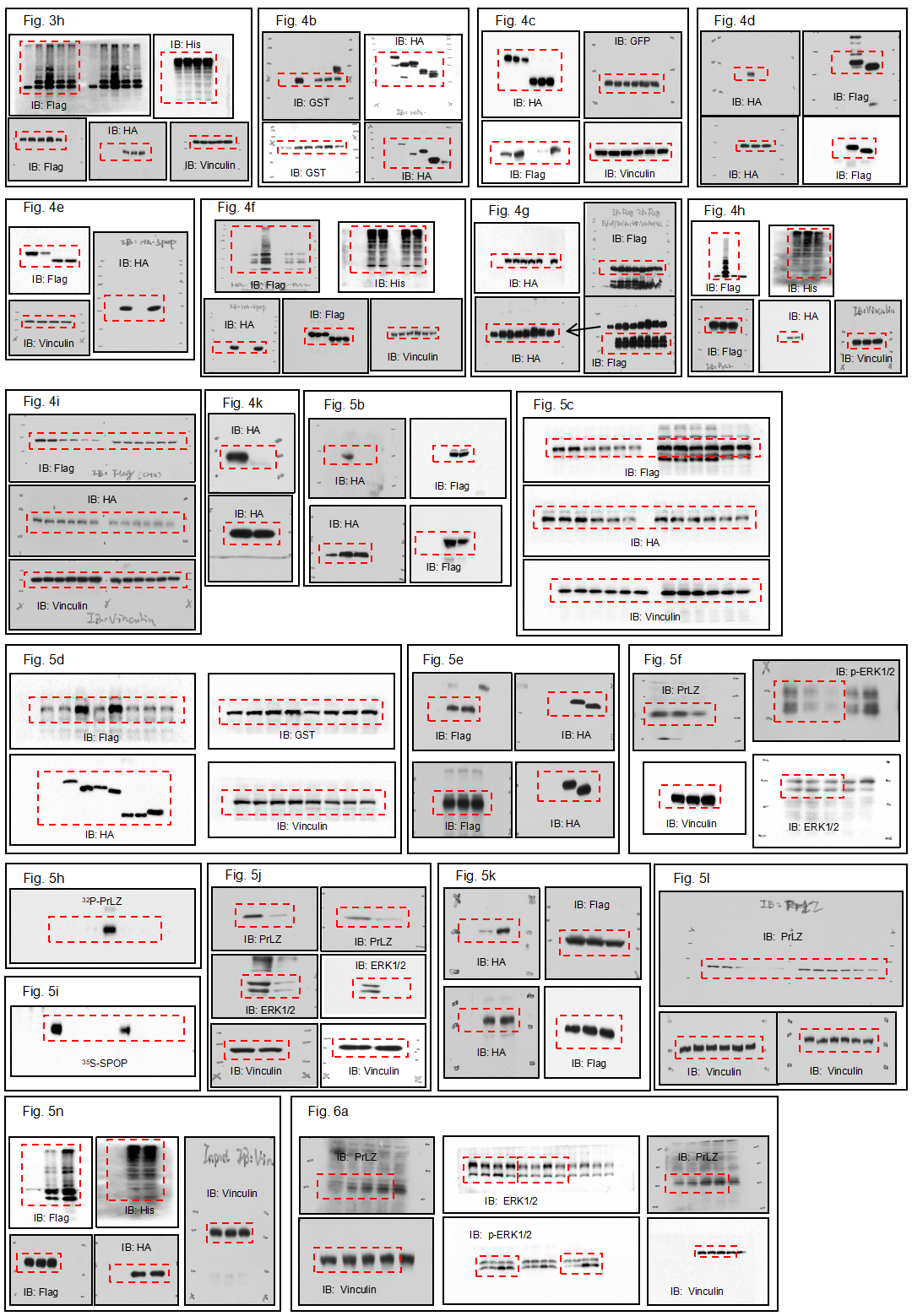
**

**Supplementary Figure S10**

**
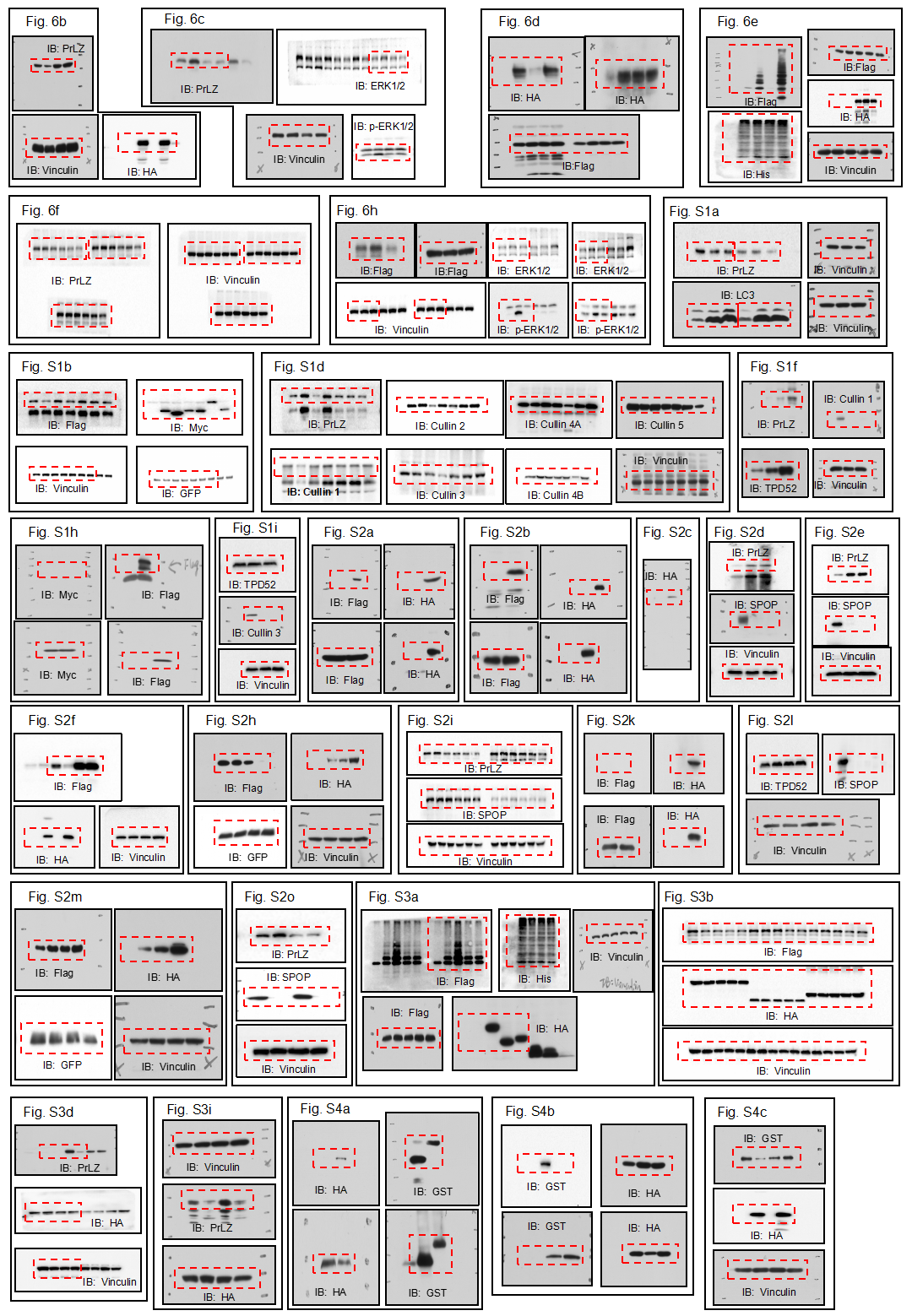
**

**Supplementary Figure S11**

**
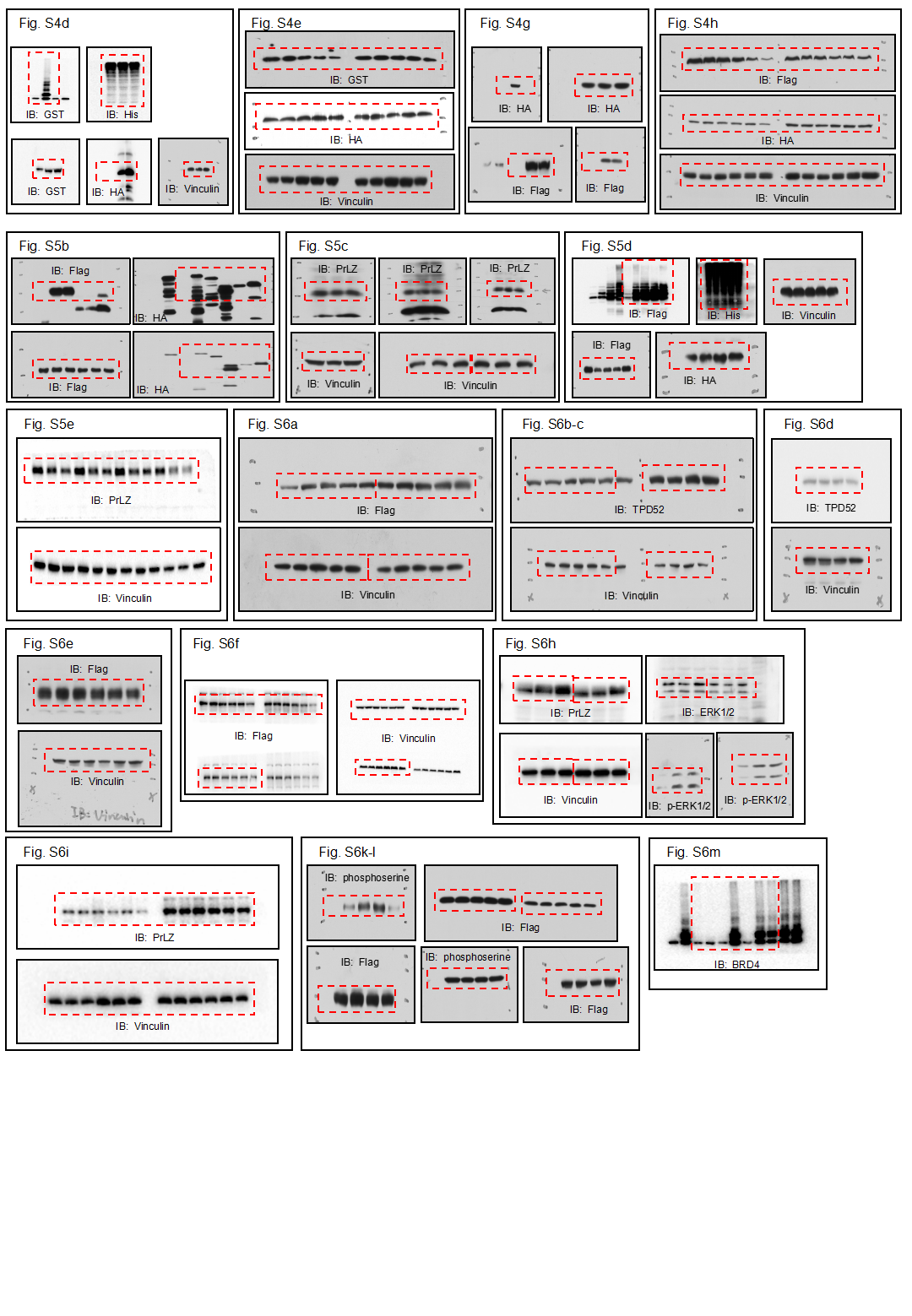
**
