# Supplementary material for: ERK1/2 inhibits Cullin 3/SPOP-mediated PrLZ ubiquitination and degradation to modulate prostate cancer progression
Source: Cell Death Differ. 2022 Feb 22;29(8):1611–24. doi: 10.1038/s41418-022-00951-y (PMC9345960; doi:10.1038/s41418-022-00951-y)
Supplement: Supplementary file 2 — Supplementary Figure legends and Table [file 41418_2022_951_MOESM2_ESM.doc]

Supplementary Figure Legends and Table for

**ERK1/2 inhibits Cullin 3/SPOP-mediated PrLZ ubiquitination and degradation to modulate prostate cancer progression**

Yizeng Fan^1,3^, Tao Hou^1,3^, Weichao Dan^1,3^, Yasheng Zhu^2,3^, Bo Liu^1^, Yi Wei^1^, Zixi Wang^1^, Yang Gao^1^, Jin Zeng^1,*^, and Lei Li^1,*^

^1^ Department of Urology, The First Affiliated Hospital of Xi'an Jiaotong University, 710061, Xi'an, P. R. China.

^2^ Department of Urology, Shanghai Changhai Hospital, Second Military Medical University, 200433, Shanghai, P.R. China.

^3^ These authors contributed equally to this work.

^*^Correspondence and requests for materials should be addressed to J.Z. (zengjin1984@126.com); L.L. (E-mail: [lilydr@163.com)](mailto:lilydr@163.com)).

**This file includes:**

Supplementary Figure Legends S1-S7

Supplementary Table 1

**Supplementary Figure S1. Cullin 1 and Cullin 3-based E3 ubiquitin ligases negatively regulate PrLZ protein stability. a** Immunoblot (IB) analysis of whole-cell lysates (WCL) derived from C4-2 cells treated with increasing dose of chloroquine (CQ) or NH_4_Cl for 12 hours. IB analysis of WCL derived from 293T cells transfected with Flag-PrLZ and indicated Myc-tagged Cullins (**b**) and from C4-2 cells stably expressing indicated shCullins as well as shScr as a negative control (**d**). EV, empty vector as a negative control. Scr, Scramble. **c.e** Quantification of the band intensities in panel b and panel d, respectively. PrLZ bands were normalized to vinculin. The control group was normalized to 1 and all other groups were compared to control. An unpaired *t*-test was performed to compare the control and all other groups based on the values from three independent experiments. Error bars represented standard deviation (S.D.) **P*＜0.05. **f** IB analysis of WCL derived from C4-2 cells stably expressing shCullin 1 as well as shScr as a negative control. Scr, Scramble. **g** A schematic diagram representing the PrLZ and TPD52 structural domains. **h** IB analysis of WCL and anti-Flag immunoprecipitates (IPs) derived from 293T cells transfected with Flag-TPD52 and Myc-Cullin 3. EV, empty vector. **i** IB analysis of WCL derived from C4-2 cells stably expressing shCullin 3 as well as shScr as a negative control. Scr, Scramble.

**Supplementary Figure S2. SPOP specifically interacts with and promotes PrLZ poly-ubiquitination and degradation. a** IB analysis of WCL and anti-HA IPs derived from 293T cells transfected with HA-SPOP and Flag-PrLZ. EV, empty vector. **b** IB analysis of WCL and anti-HA IPs derived from 293T cells transfected with HA-PrLZ and Flag-SPOP. EV, empty vector. **c** GST pull-down assay revealed the direct interaction between PrLZ and SPOP. The upper panel presents the result of IB by using the antibody against HA, and the lower coomassie blue staining showing the gels for purified proteins. **d** IB analysis of WCL derived from C4-2 cells stably expressing shSPOP as well as shScr as a negative control. Scr, Scramble. **e** IB analysis of WCL derived from C4-2 cells stably expressing siSPOP as well as siNC as a negative control. NC, negative control. **f** IB analysis of WCL derived from 293T cells transfected with Flag-PrLZ and HA-SPOP. Where indicated, 20 μM MG132 was added for 6 hours before harvesting the cells. **g** Real-time PCR analysis to examine PrLZ mRNA levels after depletion of endogenous SPOP by shRNA (shSPOP). Data were shown as mean ± SD of three independent experiments. **h** IB analysis of WCL derived from 293T cells transfected with Flag-PrLZ and increasing transfection doses (0.5, 1 and 3 μg) of HA-SPOP. EV, empty vector. **i** SPOP knockdown cells (shSPOP) as well as parental C4-2 cells (shScr) were treated with 100 μg/ml cycloheximide (CHX) for the indicated time period before harvesting. Equal amounts of WCL were immunoblotted with the indicated antibodies. **j** The PrLZ protein abundance in (**i**) was quantified by ImageJ and plotted as indicated. PrLZ bands were normalized to vinculin. **k** IB analysis of WCL and anti-HA IPs derived from 293T cells transfected with Flag-TPD52 and HA-SPOP. EV, empty vector. **l** IB analysis of WCL derived from C4-2 cells with *SPOP* knockout by the CRISPR technology. Parental C4-2 cells are used as the control. **m** IB analysis of WCL derived from 293T cells transfected with Flag-TPD52 and increasing transfection dose (0.5, 1.5 and 3 μg) of HA-SPOP. **n** Mass spectrometry analysis revealed SPOP-mediated PrLZ ubiquitination at lysine residues 24, 100, and 120. **o** IB analysis of WCL derived from C4-2 cells stably expressing shSPOP or/and shPrLZ as well as shScr as a negative control. Scr, Scramble.

**Supplementary Figure S3. PCa-associated SPOP Mutants fail to interact with and promote PrLZ poly-ubiquitination and degradation. a** IB analysis of WCL and Ni-NTA pull-down products derived from PC-3 cells transfected with Flag-PrLZ, His-Ub, and HA-SPOP WT or MATH domain deletion SPOP (ΔMATH) or BTB domain deletion SPOP (ΔBTB) constructs. Where indicated, 20 μM MG132 was added for 6 hours before harvesting the cells. EV, empty vector. WT, wild type. **b** IB analysis of WCL derived from 293T cells transfected with Flag-PrLZ and HA-SPOP WT or ΔMATH SPOP or ΔBTB SPOP constructs. Where indicated, 100 μg/ml CHX was added for the indicated time period before harvesting. WT, wild type. **c** The PrLZ protein abundance in (**b**) was quantified by ImageJ and plotted as indicated. PrLZ bands were normalized to vinculin. **d** IB analysis of WCL derived from PC-3 cells stably co-expressing HA-SPOP WT or HA-SPOP F102C mutant and Flag-PrLZ. EV, empty vector. WT, wild type. **e** The growth curve of PC-3 cells stably co-expressing HA-SPOP WT or HA-SPOP F102C mutant and Flag-PrLZ. EV, empty vector. WT, wild type. **P* < 0.05. **f-h** PC-3 cells stably co-expressing HA-SPOP WT or HA-SPOP F102C mutant and Flag-PrLZ were subcutaneously injected into nude mice to establish xenograft model. Statistical analysis of the tumor volumes which were measured every three days and plotted individually **(g)**. Subcutaneous xenograft tumors formed from different groups in PC-3 cells were dissected **(f)**. Statistical analysis of the weight of the dissected xenografts tumors **(h)**. n = 5 mice per experimental group, the results indicate the mean ± S.D. ***P*＜0.01 and ^ns^*P*>0.05. EV, empty vector. WT, wild type. **i** IB analysis of WCL derived from C4-2 cells stably co-expressing SPOP-F102C or SPOP-W131G mutants and shPrLZ or shScr. Scr, Scramble control. **j** The growth curve of C4-2 cells stably co-expressing SPOP-F102C or SPOP-W131G mutants and shPrLZ or shScr. Scr, Scramble. **P* < 0.05. **k-l** Colony formation assays and quantification of C4-2 cells stably co-expressing SPOP-F102C or SPOP-W131G mutants and shPrLZ or shScr. Scr, Scramble. ***P* < 0.01.

**Supplementary Figure S4. SPOP promotes PrLZ ubiquitination and degradation through interaction with the distinctive N-terminal of PrLZ. a** IB analysis of WCL and GST pull-down precipitates derived from 293T cells transfected with GST-PrLZ amino acid (aa) 1-46 and HA-SPOP. Where indicated, 20 μM MG132 was added for 6 hours before harvesting the cells. **b** IB analysis of WCL and anti-HA IPs derived from 293T cells transfected with GST-PrLZ aa 1-46 WT or GST-PrLZ aa 1-46 S40A and HA-SPOP. Where indicated, 20 μM MG132 was added for 6 hours before harvesting the cells. EV, empty vector. WT, wild type. **c** IB analysis of WCL derived from 293T cells transfected with GST-PrLZ aa 1-46 WT or GST-PrLZ aa 1-46 S40A and HA-SPOP. WT, wild type. **d** IB analysis of WCL and Ni-NTA pull-down products derived from PC-3 cells transfected with GST-PrLZ aa 1-46 WT or GST-PrLZ aa 1-46 S40A, HA-SPOP and His-Ub. Where indicated, 20 μM MG132 was added for 6 hours before harvesting the cells. WT, wild type. **e** IB analysis of WCL derived from 293T cells transfected with GST-PrLZ aa 1-46 WT or GST-PrLZ aa 1-46 S40A and HA-SPOP. Where indicated, 100 μg/ml CHX was added for the indicated time period before harvesting. WT, wild type. **f** The GST PrLZ aa 1-46 protein abundance in (**e**) was quantified by ImageJ and plotted as indicated. PrLZ bands were normalized to vinculin. **g** IB analysis of WCL and anti-Flag IPs derived from 293T cells transfected with Flag-PrLZ WT or Flag-PrLZ S40R and HA-SPOP. Where indicated, 20 μM MG132 was added for 6 hours before harvesting the cells. EV, empty vector. WT, wild type. **h** IB analysis of WCL derived from 293T cells transfected with Flag-PrLZ WT, Flag-PrLZ S40R and HA-SPOP. Where indicated, 100 μg/ml CHX was added for the indicated time period before harvesting. WT, wild type. **i** The Flag-PrLZ protein abundance in (**h**) was quantified by ImageJ and plotted as indicated. PrLZ bands were normalized to vinculin.

**Supplementary Figure S5. ERK1/2 mediated phosphorylation of PrLZ at Ser40 stabilizes PrLZ through disrupting its binding with SPOP.**

**a** Quantification of the band intensities in **(Fig. 5c)**.The PrLZ protein abundance in (**Fig. 5c**) was quantified by ImageJ and plotted as indicated. PrLZ bands were normalized to vinculin. **b** IB analysis of WCL and anti-HA IPs derived from 293T cells transfected with Flag-PrLZ and indicated kinases constructs. 30 hours post-transfection, cells were treated with 20 μM MG132 for 6 hours before harvesting. EV, empty vector. **c** IB analysis of WCL derived from C4-2 cells treated with Compound C (5 and 10 μM) or CHIR99021 (10 and 20 μM) for 24 hours or palbociclib (1 and 3 μM) for 48 hours. **d** IB analysis of WCL and Ni-NTA pull-down products derived from PC-3 cells transfected with Flag-PrLZ, HA-SPOP and His-Ub. Cells were treated with indicated inhibitors. Where indicated, 20 μM MG132 was added for 6 hours before harvesting the cells. WT, wild type. **e** IB analysis of WCL derived from C4-2 cells treated with indicated inhibitors. Where indicated, 100 μg/ml CHX was added for the indicated time period before harvesting. **f** The PrLZ protein abundance in (**e**) was quantified by ImageJ and plotted as indicated. PrLZ bands were normalized to vinculin. **g** The growth curve of C4-2 cells stably expressing PrLZ-WT or PrLZ-S40D mutant treated with 3 μM SCH772984. **P* < 0.05. **h-i** Colony formation assays and quantification of C4-2 cells stably expressing PrLZ-WT or PrLZ-S40D mutant treated with 3 μM SCH772984. ***P* < 0.01.

**Supplementary Figure S6. IL-6 protects PrLZ from degradation through activating ERK1/2. a** IB analysis of WCL derived from 293T cells transfected with Flag-PrLZ WT or Flag-PrLZ S40A mutant. Cells were then treated with different concentration of IL-6 (10, 25, 50 and 100 ng/ml) for 48 hours. **b** IB analysis of WCL derived from C4-2 cells treated with different concentration of IL-6 (10, 25, 50 and 100 ng/ml) for 48 hours. **c** IB analysis of WCL derived from C4-2 cells treated with IL-6 (50 ng/ml) for indicated time points. **d** IB analysis of WCL derived from C4-2 cells treated with IL-6 (50 ng/ml) or/and SCH772984 (3 μM). **e** IB analysis of WCL derived from 293T cells transfected with Flag-PrLZ S40D mutant. Cells were then treated with different concentration of IL-6 (50 and 100 ng/ml) or SCH772984 (1 and 3 μM). **f** IB analysis of WCL derived from 293T cells transfected with Flag-PrLZ S40D. Cells were then treated with IL-6 (50 ng/ml) or SCH772984 (3 μM). Where indicated, 100 μg/ml CHX was added for the indicated time period before harvesting. **g** The PrLZ protein abundance in (**f**) was quantified by ImageJ and plotted as indicated. PrLZ bands were normalized to vinculin. **h** IB analysis of WCL derived from C4-2 and 22Rv1 cells treated with different concentration of EGF (5 and 10 ng/ml) for 24 hours. **i** IB analysis of WCL derived from C4-2 cells treated with EGF (10 ng/ml). Where indicated, 100 μg/ml CHX was added for the indicated time period before harvesting. **j** The PrLZ protein abundance in (**i**) was quantified by ImageJ and plotted as indicated. PrLZ bands were normalized to vinculin. **k-l** IB analysis of WCL and anti-Flag IPs derived from 293T cells transfected with Flag-PrLZ WT **(k)** and Flag-PrLZ S40D **(l)**. Cells were treated with IL-6 (50 ng/ml), EGF (10 ng/ml) or SCH772984 (3 μM). 30 hours post-transfection, cells were treated with 20 μM MG132 for 6 hours before harvesting. **m** *In vitro* ubiquitination assay of His-BRD4-N (aa 1-500) by SPOP were performed together with reconstituted SPOP-CUL3-RBX1 E3 ligase complex with E1, E2, Ub.

**Supplementary Figure S7. *In vitro* and *in vivo* effects of IL-6 and SCH772984 on C4-2 cells. a** The growth curve of C4-2 cells treated with IL-6 (50 ng/ml) or/and SCH772984 (3 μM). **P* < 0.05. **b-c** Colony formation assays and quantification of C4-2 cells treated with IL-6 (50 ng/ml) or/and SCH772984 (3 μM). ***P* < 0.01. **d-f** C4-2 cells were subcutaneously injected into nude mice which received IL-6 (100 ng per mouse) or/and SCH772984 (50mg/kg, daily) treatment. Statistical analysis of the tumor volumes which were measured every three days and plotted individually **(d)**. Subcutaneous xenograft tumors formed from different groups in C4-2 cells were dissected **(e)**. Statistical analysis of the weights of the dissected xenografts tumors **(f)**. n = 6 mice per experimental group, the results indicated the mean ± S.D. ^**^*P*＜0.01.

Supplementary Table 1

Clinical parameters of PCa patients

| NO. | SPOP status | p-ERK1/2 IHC intensity | PrlZ IHC intensity | Age | Preoperative PSA level (ng/ml) | Prostatectomy specimen Gleason score | Pathologic tumor stage |
| --- | --- | --- | --- | --- | --- | --- | --- |
| 1 | wild type | 0 | 1 | 65 | 14.141 | 7 | pT2 |
| 2 | wild type | 3 | 3 | 64 | 52.23 | 9 | pT3 |
| 3 | wild type | 0 | 0 | 78 | 8.01 | 7 | pT2 |
| 4 | Mutation F102Y | 3 | 2 | 68 | 9.85 | 10 | pT3 |
| 5 | wild type | 0 | 1 | 69 | 12.8 | 7 | pT2 |
| 6 | Mutation F125C | 3 | 3 | 68 | 9.68 | 7 | pT4 |
| 7 | wild type | 2 | 2 | 73 | 23.62 | 7 | pT3 |
| 8 | wild type | 1 | 3 | 75 | 18.472 | 7 | pT2 |
| 9 | wild type | 1 | 1 | 70 | 8.68 | 7 | pT2 |
| 10 | wild type | 1 | 1 | 74 | 63.828 | 9 | pT3 |
| 11 | wild type | 3 | 2 | 76 | 11.557 | 6 | pT2 |
| 12 | wild type | 1 | 2 | 70 | 29.5 | 7 | pT3 |
| 13 | wild type | 0 | 0 | 55 | 87.11 | 7 | pT4 |
| 14 | wild type | 0 | 0 | 75 | 8 | 6 | pT2 |
| 15 | wild type | 0 | 0 | 77 | 3.5 | 6 | pT2 |
| 16 | Mutation F133L | 2 | 1 | 61 | 7.9 | 9 | pT2 |
| 17 | wild type | 2 | 3 | 67 | 39.6 | 7 | pT3 |
| 18 | wild type | 3 | 3 | 78 | 35.43 | 8 | pT2 |
| 19 | wild type | 0 | 1 | 56 | 5.963 | 9 | pT3 |
| 20 | wild type | 1 | 0 | 68 | 7.489 | 6 | pT2 |
| 21 | Mutation W131G | 2 | 2 | 70 | 31.19 | 6 | pT2 |
| 22 | Mutation F102C | 0 | 1 | 60 | 9.943 | 7 | pT3 |
| 23 | Mutation I8M | 0 | 1 | 70 | 10.1 | 9 | pT2 |
| 24 | Mutation F133L | 0 | 0 | 73 | 21.788 | 7 | pT2 |
| 25 | Mutation F133L | 3 | 3 | 66 | 70.965 | 9 | pT3 |
| 26 | Mutation G38W | 0 | 1 | 67 | 39.83 | 9 | pT2 |
| 27 | Mutation F133L | 2 | 2 | 76 | 9.87 | 9 | pT2 |
| 28 | wild type | 0 | 0 | 61 | 14.865 | 8 | pT3 |
| 29 | wild type | 0 | 0 | 60 | 10.673 | 7 | pT2 |
| 30 | Mutation W131G | 2 | 1 | 51 | 27.04 | 7 | pT3 |
| 31 | wild type | 1 | 1 | 75 | 12.568 | 7 | pT2 |
| 32 | wild type | 1 | 1 | 62 | 7.95 | 7 | pT2 |
| 33 | wild type | 1 | 1 | 66 | 6.777 | 7 | pT2 |
| 34 | wild type | 1 | 2 | 60 | 10.088 | 6 | pT2 |
| 35 | wild type | 0 | 0 | 70 | 90.81 | 9 | pT3 |
| 36 | wild type | 1 | 1 | 72 | 25.81 | 6 | pT2 |
| 37 | wild type | 1 | 2 | 71 | 51.647 | 9 | pT2 |
| 38 | wild type | 2 | 3 | 72 | 7.402 | 7 | pT3 |
| 39 | wild type | 2 | 2 | 74 | 18.74 | 7 | pT2 |
| 40 | wild type | 0 | 1 | 55 | 7.441 | 7 | pT2 |
| 41 | wild type | 3 | 1 | 80 | 16.542 | 8 | pT2 |
| 42 | wild type | 0 | 1 | 61 | 14.58 | 8 | pT2 |
| 43 | wild type | 0 | 1 | 55 | 30.822 | 6 | pT2 |
| 44 | wild type | 0 | 0 | 73 | 13.11 | 7 | pT2 |
| 45 | Mutation F133V | 3 | 1 | 67 | 13.946 | 7 | pT2 |
| 46 | wild type | 0 | 1 | 69 | 2.522 | 7 | pT2 |
| 47 | wild type | 1 | 1 | 72 | 29.957 | 7 | pT2 |
| 48 | wild type | 0 | 0 | 69 | 4.5 | 7 | pT2 |
| 49 | wild type | 0 | 0 | 72 | 21.91 | 6 | pT2 |
| 50 | wild type | 0 | 1 | 56 | 4.004 | 7 | pT2 |
| 51 | Mutation F102S | 0 | 1 | 72 | 48.13 | 8 | pT4 |
| 52 | wild type | 1 | 1 | 68 | 11.52 | 7 | pT2 |
| 53 | wild type | 1 | 2 | 64 | 10.3 | 7 | pT2 |
| 54 | wild type | 0 | 1 | 74 | 39.5 | 7 | pT2 |
| 55 | wild type | 2 | 2 | 72 | 19.76 | 10 | pT3 |
| 56 | wild type | 0 | 1 | 68 | 47.28 | 9 | pT3 |
| 57 | wild type | 0 | 0 | 67 | 7.66 | 9 | pT3 |
| 58 | wild type | 2 | 3 | 58 | 24.08 | 9 | pT4 |
| 59 | wild type | 0 | 1 | 77 | 4.618 | 8 | pT2 |
| 60 | wild type | 2 | 2 | 70 | 11.556 | 9 | pT2 |
| 61 | wild type | 0 | 1 | 72 | 9 | 6 | pT2 |
| 62 | wild type | 0 | 1 | 70 | 14.2 | 9 | pT2 |
| 63 | wild type | 0 | 1 | 69 | 19.7 | 7 | pT2 |
| 64 | wild type | 1 | 1 | 59 | 5.257 | 8 | pT2 |
| 65 | wild type | 1 | 3 | 60 | 20.45 | 9 | pT2 |
| 66 | wild type | 3 | 1 | 63 | 17.93 | 8 | pT2 |
| 67 | wild type | 1 | 3 | 75 | 1.29 | 9 | pT3 |
| 68 | wild type | 1 | 1 | 63 | 8.61 | 7 | pT2 |
| 69 | wild type | 2 | 2 | 69 | 25.723 | 8 | pT3 |
| 70 | wild type | 1 | 3 | 46 | 5.964 | 7 | pT3 |
| 71 | wild type | 0 | 0 | 60 | 164.9 | 9 | pT4 |
| 72 | wild type | 0 | 2 | 64 | 17.94 | 8 | pT2 |
| 73 | wild type | 0 | 0 | 74 | 8.38 | 7 | pT2 |
| 74 | wild type | 1 | 2 | 75 | 16.75 | 9 | pT2 |
| 75 | wild type | 3 | 3 | 79 | 100 | 9 | pT4 |
| 76 | wild type | 1 | 2 | 69 | 7.89 | 8 | pT2 |
| 77 | wild type | 1 | 1 | 77 | 8.32 | 8 | pT3 |
| 78 | wild type | 2 | 2 | 63 | 21.674 | 7 | pT3 |
| 79 | Mutation Q9E | 2 | 2 | 70 | 15.312 | 8 | pT3 |
| 80 | wild type | 2 | 2 | 64 | 15.8 | 6 | pT3 |
| 81 | wild type | 3 | 3 | 88 | 9.93 | 9 | pT3 |
| 82 | wild type | 2 | 2 | 71 | 21.964 | 6 | pT2 |
| 83 | wild type | 1 | 1 | 69 | 7.64 | 9 | pT3 |
| 84 | wild type | 0 | 1 | 64 | 19.673 | 8 | pT2 |
| 85 | wild type | 1 | 2 | 68 | 44.457 | 7 | pT3 |
| 86 | Mutation E39X | 3 | 3 | 58 | 16.833 | 7 | pT3 |
| 87 | wild type | 2 | 3 | 75 | 47.91 | 8 | pT3 |
| 88 | Mutation I39S | 3 | 3 | 65 | 18.91 | 7 | pT2 |
| 89 | Mutation S80G | 2 | 3 | 68 | 78.128 | 9 | pT3 |
| 90 | wild type | 0 | 1 | 74 | 17.361 | 7 | pT2 |
| 91 | wild type | 0 | 2 | 68 | 31.23 | 7 | pT2 |
| 92 | wild type | 0 | 2 | 65 | 23.39 | 7 | pT2 |
| 93 | wild type | 0 | 2 | 66 | 55.98 | 9 | pT2 |
| 94 | Mutation I39S | 1 | 3 | 64 | 9.587 | 7 | pT3 |
| 95 | Mutation I8N | 1 | 3 | 74 | 14.536 | 7 | pT3 |
| 96 | wild type | 3 | 3 | 78 | 23.494 | 7 | pT3 |
| 97 | wild type | 0 | 1 | 74 | 15.64 | 7 | pT2 |
| 98 | Mutation I39N | 0 | 2 | 67 | 38.03 | 9 | pT3 |
| 99 | wild type | 0 | 2 | 72 | 13.35 | 9 | pT3 |
| 100 | wild type | 1 | 1 | 75 | 7.6 | 7 | pT3 |
| 101 | wild type | 1 | 2 | 80 | 11.428 | 7 | pT3 |
| 102 | wild type | 0 | 1 | 64 | 6.517 | 7 | pT2 |
| 103 | wild type | 0 | 0 | 64 | 5.473 | 7 | pT2 |
| 104 | wild type | 1 | 1 | 78 | 19.104 | 7 | pT2 |
| 105 | wild type | 0 | 2 | 71 | 32.26 | 9 | pT3 |
| 106 | Mutation I39S | 0 | 2 | 67 | 11.36 | 8 | pT3 |
| 107 | wild type | 0 | 0 | 62 | 37.88 | 7 | pT2 |
| 108 | wild type | 0 | 1 | 69 | 95.3 | 9 | pT3 |
| 109 | wild type | 0 | 1 | 69 | 23.46 | 9 | pT3 |
| 110 | wild type | 0 | 3 | 70 | 37.19 | 9 | pT3 |
| 111 | wild type | 1 | 2 | 65 | 8.7 | 7 | pT3 |
| 112 | wild type | 1 | 2 | 60 | 19.312 | 8 | pT2 |
| 113 | wild type | 1 | 2 | 60 | 1161.92 | 8 | pT3 |
| 114 | Mutation I39S | 0 | 1 | 79 | 100 | 9 | pT3 |
| 115 | wild type | 1 | 1 | 68 | 9.11 | 7 | pT3 |
| 116 | wild type | 1 | 1 | 76 | 9.44 | 8 | pT2 |
| 117 | wild type | 1 | 3 | 72 | 17.01 | 7 | pT2 |
| 118 | wild type | 1 | 1 | 74 | 11.35 | 7 | pT3 |
| 119 | wild type | 1 | 1 | 57 | 22 | 8 | pT2 |
| 120 | wild type | 1 | 2 | 63 | 8.062 | 9 | pT2 |
| 121 | wild type | 0 | 1 | 66 | 30.56 | 7 | pT3 |
| 122 | wild type | 1 | 1 | 70 | 21.78 | 7 | pT2 |
| 123 | wild type | 1 | 1 | 59 | 22.88 | 9 | pT3 |
| 124 | wild type | 0 | 1 | 70 | 14 | 7 | pT2 |
| 125 | wild type | 3 | 3 | 76 | 29.74 | 9 | pT2 |
| 126 | Mutation Q40E | 3 | 3 | 78 | 34.56 | 7 | pT2 |
| 127 | wild type | 0 | 3 | 64 | 10.3 | 7 | pT3 |
| 128 | wild type | 1 | 1 | 60 | 1.86 | 8 | pT2 |
