# Supplementary material for: ERK1/2 inhibits Cullin 3/SPOP-mediated PrLZ ubiquitination and degradation to modulate prostate cancer progression
Source: Cell Death Differ. 2022 Feb 22;29(8):1611–24. doi: 10.1038/s41418-022-00951-y (PMC9345960; doi:10.1038/s41418-022-00951-y)
Supplement: Supplementary file 3 — marked manuscripts [file 41418_2022_951_MOESM3_ESM.docx]

**ERK1/2 inhibits Cullin 3/SPOP-mediated PrLZ ubiquitination and degradation to modulate prostate cancer progression**

Yizeng Fan^1,3^, Tao Hou^1,3^, Weichao Dan^1,3^, Yasheng Zhu^2,3^, Bo Liu^1^, Yi Wei^1^, Zixi Wang^1^, Yang Gao^1^, Jin Zeng^1,*^, and Lei Li^1,*^

^1^ Department of Urology, The First Affiliated Hospital of Xi'an Jiaotong University, 710061, Xi'an, P. R. China.

^2^ Department of Urology, Shanghai Changhai Hospital, Second Military Medical University, 200433, Shanghai, P.R. China.

^3^ These authors contributed equally to this work.

^*^ Correspondence and requests for materials should be addressed to J.Z. (zengjin1984@126.com); L.L. (E-mail: [lilydr@163.com)](mailto:lilydr@163.com)).

**Running title:** CULLIN 3/SPOP DEGRADES PRLZ

**Abstract**

The gene encoding the E3 ubiquitin ligase substrate-binding adaptor SPOP is frequently mutated in prostate cancer (PCa), but how SPOP functions as a tumor suppressor and contribute to PCa pathogenesis remains poorly understood. Prostate Leucine Zipper (*PrLZ*) serves as a prostate-specific and androgen-responsive gene, which plays a pivotal role in the malignant progression of PCa. However, the upstream regulatory mechanism of PrLZ protein stability and its physiological contribution to PCa carcinogenesis remain largely elusive. Here we report that PrLZ can be degraded by SPOP. PrLZ abundance is elevated in SPOP-mutant expressing PCa cell lines and patient specimens. Meanwhile, ERK1/2 might regulate SPOP-mediated PrLZ degradation through phosphorylating PrLZ at Ser40, which blocks the interaction between SPOP and PrLZ. In addition, we identify IL-6 might act as an upstream PrLZ degradation regulator *via* promoting its phosphorylation by ERK1/2, leading to its impaired recognition by SPOP. Thus, our study reveals a novel SPOP substrate PrLZ which might be controlled by ERK1/2-mediated phosphorylation, thereby facilitating to explore novel drug targets and improve therapeutic strategy for PCa.

**Keywords:** PrLZ, SPOP, Phosphorylation, ERK1/2, IL-6

**Introduction**

Prostate cancer (PCa) is the second leading cause of death in male cancer patients, accounting for about 26 percent of new cancer cases in western countries[1]. Accumulating evidence indicates that the activation of oncogenes is responsible for PCa initiation and progression. Prostate Leucine Zipper (*PrLZ*), a member of the tumor protein D52 (TPD52) family, is an important prostate-specific and androgen-responsive oncogene involved in the malignant progression of PCa[2]. Our previous studies have demonstrated that PrLZ over-expression in PCa facilitates PCa progression largely by promoting cell growth, chemotherapy resistance, cell migration and invasion[3, 4, 5, 6]. We also identified that, instead of being degraded through lysosome, PrLZ regulated chaperone-mediated autophagy pathway by directly interacting with Hsc70[7]. However, the upper regulatory mechanism of PrLZ, especially the physiological E3 ubiquitin ligase(s) that governs PrLZ protein stability still remains largely unknown.

Systematic sequencing studies reveal that recurrent somatic mutation in *SPOP* (Speckle-type POZ protein), a substrate-interacting adaptor for the Cullin 3-based E3 ubiquitin ligase complexes with a mutation rate of 10% to 15%, is a key molecular feature of PCa[8, 9]. Intriguingly, these SPOP mutations are currently clustered in the substrate-binding MATH domain[10]. PCa-associated missense mutations in the MATH domain of SPOP disrupt substrate binding and ubiquitination, leading to upregulated oncogenic substrate levels and increased PCa cell proliferation and invasion, indicating the tumor-suppressive role of SPOP in PCa. In PCa, oncoproteins including AR[11], BRD4[12, 13], SRC-3[14], DEK[15], TRIM24[16], PD-L1[17, 18], 17βHSD4[19], HIPK2[20], ZMYND11[21] and ERG[22] are well-known substrates of SPOP. Whereas, SPOP is over-expressed and mislocalized in kidney cancer, acting as an oncogenic role [23, 24]. The oncogenic function of SPOP can be inhibited by small molecules that can inhibit the SPOP-substrate protein interaction[25]. Meanwhile, miR-520/372/373 family can inhibit renal cell carcinoma progression through suppressing SPOP protein expression[26]. Considering the tissue and cellular context dependent role of SPOP in tumorigenesis, here we will mainly focus on uncovering the tumor suppressor role of SPOP in PCa.

In this study, we report that SPOP partially modulates PCa progression via promoting PrLZ ubiquitination and degradation. Meanwhile, ERK1/2 activation phosphorylates PrLZ at Ser40 and inhibits the PrLZ degradation by blocking the binding of PrLZ to SPOP. Thus, our study reveals a possible phosphorylation-dependent regulatory mechanism involved in regulation of PrLZ stability dictated by SPOP-induced degradation, potentially opening new therapeutic avenues for PCa treatment.

**Results**

**Cullin 1 and Cullin 3-based E3 ubiquitin ligases negatively regulate PrLZ protein stability.**

PrLZ is over-expressed in human PCa tissues and contributes to the malignant progression of PCa[2], whereas the regulatory mechanism of PrLZ protein stability remains elusive. Our study indicated that MG132, a peptide aldehyde proteasome inhibitor, increased the protein level of PrLZ (Fig. 1a and b) in C4-2 and 22Rv1 cells. However, lysosome inhibitor chloroquine or NH_4_Cl failed to increase PrLZ protein level (Fig. S1a), suggesting the involvement of ubiquitin-mediated pathways in controlling PrLZ stability. Notably, treatment with MLN4924, an inhibitor of Cullin-Ring ligases (CRLs) by blocking cullin neddylation[27], elevated endogenous PrLZ protein level (Fig. 1a and 1b), indicating the negative regulation of PrLZ stability through CRL(s).

To screen the potential Cullin-based E3 ubiquitin ligases responsible for PrLZ destruction, we detected the interaction between PrLZ and members of the Cullin family. As shown in Fig. 1c and 1d, Cullin 3 and Cullin 1, instead of other Cullin family members (Cullin 2, 4A, 4B and 5), interacted with PrLZ. Overexpression of Cullin 1 and Cullin 3 markedly decreased exogenous PrLZ protein level (Fig. S1b and S1c), while depletion of Cullin 1 and Cullin 3 increased PrLZ protein level (Fig. 1e, S1d, S1e and S1f). Interestingly, depletion of Cullin 1 by shRNAs could also increase the amount of TPD52 protein (Fig. S1f). Given the extensive evidence linking Cullin 3 to PCa, we thus just focused on Cullin 3 in this study. Overexpression of Cullin 3 promoted both endogenous and exogenous PrLZ protein degradation in a dose-dependent manner (Fig. 1f and 1g). Consistently, Cullin 3 overexpression promoted PrLZ protein ubiquitination (Fig. 1h) and Cullin 3 depletion significantly extended the half-life of PrLZ protein in C4-2 cells (Fig. 1i and 1j).

Since PrLZ shared different N-terminal domain with TPD52 protein (Fig. S1g), we further explored the relationship between Cullin 3 and TPD52. Unexpectedly, TPD52 failed to interact with Cullin 3 (Fig. S1h) and Cullin 3 depletion had no effects on TPD52 protein level (Fig. S1i). Collectively, these results demonstrate that Cullin 1 and Cullin 3-based E3 ubiquitin ligases negatively regulate PrLZ protein stability in PCa.

**SPOP interacts with and promotes PrLZ poly-ubiquitination and degradation.**

Substrate recruiting adaptor proteins are essential for Cullin 3-based E3 ubiquitin ligases to recognize downstream substrates. We then found that both endogenous and exogenous SPOP, rather than other Cullin 3-based E3 ligase adaptor proteins including KLHL2, KLHL3, KLHL22, KLHL37, or PLZF, interacted with PrLZ (Fig. 2a and 2b, Fig. S2a and S2b). Furthermore, pull-down assay revealed a direct interaction between SPOP and PrLZ (Fig. 2c and S2c). Either CRISPR/Cas9-mediated knockout or shRNAs/siRNAs-mediated knockdown of SPOP led to a marked increase of PrLZ as well as other identified SPOP substrates, including AR and TRIM24 (Fig. 2d and 2e, Fig. S2d and S2e). Meanwhile, overexpression of SPOP promoted PrLZ protein degradation in a dose-dependent manner (Fig. 2f). Importantly, SPOP-mediated degradation of PrLZ could be blocked by MG132 (Fig. S2f) and SPOP deletion had no effects on PrLZ mRNA level (Fig. S2g), indicating that SPOP regulated PrLZ abundance through the ubiquitin-proteasome pathway. Consistently, SPOP overexpression enhanced PrLZ protein ubiquitination (Fig. 2i and S2h) and SPOP depletion significantly extended the half-life of PrLZ protein in C4-2 cells (Fig. 2g and 2h, Fig. S2i and S2j). Additionally, TPD52 was unable to bind with SPOP (Fig. S2k) and SPOP depletion showed no effect on TPD52 protein stability (Fig. S2l and S2m).

Next, we sought to determine the ubiquitin chain linkage type(s) that was generated on PrLZ by SPOP. Wild type (WT) ubiquitin and single lysine residue-only ubiquitin (K6-, K11-, K27-, K29-, K33-, K48-, and K63-ubiquitin) were overexpressed in 293T cells. We found that only K48-ubiquitin promoted SPOP-mediated PrLZ polyubiquitination (Fig. 2j). Meanwhile, we mutated each of the lysine residues on ubiquitin (K to R) to test their individual effects on PrLZ polyubiquitination in SPOP-overexpressing 293T cells. Although K6R-, K11R-, K27R-, K29R-, K33R-, and K63R-ubiquitin still triggered SPOP-mediated PrLZ polyubiquitination, the K48R-ubiquitin inhibited chain formation on PrLZ, which was similar to the results obtained with the 7KR-ubiquitin titrations (Fig. 2k). To determine the lysine residues site(s) of PrLZ that were ubiquitinated by SPOP, we applied gel electrophoresis to separate the *in vivo* ubiquitination reactions (Flag-tagged PrLZ, HA-tagged SPOP and ubiquitin) and stained the resulting gel. Trypsin digestion of the band corresponding to ubiquitinated PrLZ followed by liquid chromatography-tandem mass spectrometry (LC-MS-MS) analysis revealed the ubiquitination sites at lysine residues 24, 100, and 120. We then mutated individual lysine residues (K24A, K100A and K120A) in PrLZ, and we found that mutation of K24 significantly decreased SPOP-mediated polyubiquitination of PrLZ (Fig. 2l and S2n). Thus, our data identified the formation of a K48-linked polyubiquitin chain on PrLZ K24 residue mediated by SPOP. Functionally, depletion of PrLZ largely attenuated the enhanced cell proliferation, migration and invasion in SPOP-depleted cells (Figure 2m-q and Figure S2o).

Overall, these results indicate that SPOP may inhibit cell proliferation and migration partially through promoting PrLZ poly-ubiquitination and degradation.

**PCa-associated SPOP Mutants fail to interact with and promote PrLZ poly-ubiquitination and degradation.**

Structurally, SPOP is comprised of an N-terminal MATH domain for substrate interaction and a C-terminal BTB domain for Cullin 3 binding (Fig. 3a). As showed in Figure 3b, SPOP without MATH domain failed to interact with PrLZ. Further studies indicated that both the MATH domain and BTB domain were required for SPOP-mediated PrLZ ubiquitination and degradation (Fig. 3c and Fig. S3a-c).

Most of the PCa-associated SPOP mutations, such as Y87C, F102C, W131G and F133V, are assembled in the MATH domain and exhibit attenuated substrate binding ability [28] (Fig. 3a). We then detected the interaction between SPOP mutants and PrLZ using immunoprecipitation assay. As shown in Figure 3d, SPOP Y87C, F102C, W131G and F133V mutants failed to interact with PrLZ. Moreover, cancer-derived SPOP mutants failed to decrease endogenous PrLZ protein abundance comparing to SPOP WT (Fig. 3e), thereby incapable of affecting the half-life (Fig. 3f and 3g) and ubiquitination (Fig. 3h) of PrLZ protein.

Functionally, PrLZ could enhance cell proliferation in SPOP F102C over-expressing PC-3 cells (Fig. S3d and S3e). Furthermore, PrLZ significantly promoted the growth of SPOP F102C mutation xenografts and SPOP WT impeded PrLZ-mediated pro-tumor effects *in vivo* (Figure S3f-h). Importantly, depletion of PrLZ decreased cell proliferation and colony formation ability in cells expressing PCa-associated SPOP mutants (Fig. S3i-l). Consistently, depletion of PrLZ significantly suppressed the growth of xenografts with PCa-associated SPOP mutations *in vivo* (Fig. 3i-k).

The clinical relevance of the relationship between SPOP and PrLZ in primary human PCa was then investigated. 23 cases with SPOP-mutant and 105 cases with SPOP-WT were identified through large-scale sequencing. As shown in Figure 3l and 3m, SPOP-mutant tumors exhibited stronger PrLZ staining compared with SPOP-WT tumors. Taken together, the results showed that PCa-associated SPOP mutants failed to interact with PrLZ and resulted in accumulation of PrLZ *in vitro* and *in vivo*.

**SPOP promotes PrLZ ubiquitination and degradation through interaction with the distinctive N-terminal of PrLZ.**

We then aimed to determine the potential regions of PrLZ that interacted with SPOP. Constructs expressing different domains of PrLZ were generated (Fig. 4a). As determined by immunoprecipitation assay, amino acid 1-46 of PrLZ interacted specifically with SPOP (Fig. 4b and Fig. S4a). Additionally, compared to full length PrLZ, SPOP failed to promote PrLZ 46-224 protein degradation (Fig. 4c). Unfortunately, we didn’t find the canonical SPOP binding consensus (SBC) motif Φ-Π-S-S/T-S/T (where Φ represents a nonpolar residue; Π represents a polar residue; S represents Serine; T represents Threonine) in the N-terminal region of PrLZ. Interestingly, deletion of the amino acid 30-42 of PrLZ disrupted the binding of PrLZ to SPOP (Fig. 4d), and rendered PrLZ resistant to SPOP-meditated degradation and poly-ubiquitination (Fig. 4e and f), indicating that the amino acid 30-42 region of PrLZ might represent the potential binding motif for SPOP.

To further characterize the key site that mediated the interaction between SPOP and PrLZ, we mutated all the serine (S) or threonine (T) to alanine (A) and generated six PrLZ mutant constructs (S30A, S32A, S35A, S36A, S40A and T42A). As shown in Fig 4g and Fig. S4b, mutation of S40A significantly reduced the interaction between PrLZ and SPOP in cells. Consistently, compared with PrLZ WT, the PrLZ S40A mutant was resistant to SPOP-mediated ubiquitination and subsequent degradation (Fig. 4h-j, Fig. S4c-f). Similar to PrLZ S40A mutant, PrLZ S40R mutant also failed to interact with SPOP and was resistant to SPOP-mediated degradation (Fig. S4g-i). Moreover, SPOP bound to WT-PrLZ peptide containing ^30^SPSGNSSPPGSPT^42^, instead of S40A-mutated PrLZ (^30^SPSGNSSPPGAPT^42^) (Fig. 4k). In summary, we discovered PrLZ at serine 40 as a novel modification site in mediating the interaction and degradation of PrLZ by SPOP.

**ERK1/2-mediated phosphorylation of PrLZ at Ser40 stabilizes PrLZ through disrupting its binding with SPOP**

Although PrLZ Ser40 mutant abolished SPOP-mediated degradation, we failed to find the pathologic Ser40 mutation of PrLZ from TCGA database. These findings inspired us to explore other possible mechanisms for regulating PrLZ stability. MS analysis revealed multiple phosphorylation sites (including Ser40) on serine or threonine residues in PrLZ (Fig. 5a). To investigate the effects of Ser40 phosphorylation on the interaction between PrLZ and SPOP, we constructed a phosphorylation-mimic mutant of PrLZ (S40D). We found that the interaction between SPOP and PrLZ was totally abolished by PrLZ S40D mutant (Fig. 5b). Consistently, compared with PrLZ WT, the PrLZ S40D mutant was resistant to SPOP-mediated degradation (Fig. 5c and Fig. S5a).

We next aimed to identify the possible protein kinases responsible for the phosphorylation of PrLZ at Ser40. The amino acid sequence of PrLZ was then queried on www.phosphonet.ca to identify residues with the potential to be phosphorylated by multiple kinases including ERK1/2, CDK1, CDK2, GSK3, and CDK4. Since we have confirmed that phosphorylation of Ser40 in PrLZ may affect its protein stability, it is reasonable to interpret that kinases responsible for the phosphorylation of PrLZ should also affect the protein level of PrLZ. To determine the potential kinases, we co-expressed PrLZ and SPOP together with potential candidate kinases in 293T cells. As shown in Figure 5d, ERK1/2, instead of other kinases, significantly increased the protein level of exogenous PrLZ. Unexpectedly, not only ERK1/2 but also AMPK, GSK3β and CDK4 could bind with PrLZ (Fig. 5e and Fig. S5b). Interestingly, only ERK1/2 inhibitor SCH772984 decreased PrLZ protein level in C4-2 cells (Fig. 5f and Fig. S5c). As noted, PrLZ contains a consensus ERK1/2 phosphorylation motif PXS/TP, and the phospho-motif is evolutionally conserved in PrLZ as demonstrated by protein sequence alignment (Fig. 5g). Using *in vitro* kinase assays, we identified Ser40 as the ERK1/2 phosphorylation site (Fig. 5h). Activation of ERK1/2 phosphorylation sites impairs the interaction between PrLZ and SPOP *in vitro* (Fig. 5i). Additionally, depletion of endogenous ERK1/2 by shRNAs led to a marked decrease of PrLZ protein (Fig. 5j). Meanwhile, ERK1/2 inhibitor SCH772984 enhanced the binding between PrLZ and SPOP (Fig. 5k), and sensitized PrLZ to SPOP-meditated poly-ubiquitination and degradation (Fig. 5l-n). However, either compound c (AMPK inhibitor), CHIR99021 (GSK3β inhibitor) or palbociclib (CDK4/6 inhibitor) had no significant effects on SPOP-meditated PrLZ poly-ubiquitination and degradation (Fig. S5d-f).

In accordance with the above findings, we found that cells expressing PrLZ S40D mutants displayed enhanced cell proliferation and colony formation ability compared with cells expressing PrLZ-WT under SCH772984 treatment *in vitro* (Fig. S5g-i). Similarly, SCH772984 suppressed growth of PrLZ WT xenografts and PrLZ S40A mutation impeded SCH772984-mediated anti-tumor effects *in vivo* (Fig. 5o-q). To further evaluate the clinical correlation between p-ERK1/2 and PrLZ, we performed immunohistochemistry (IHC) staining in 128 PCa patient samples and found a strong correlation (Rho=0.596, *P*<0.01) between the two sets (Fig. 5r and 5s). Further analysis discovered that p-ERK1/2 positive tumors exhibited stronger PrLZ staining compared with p-ERK1/2 negative tumors (Fig. 5t). These results suggest that stabilization of PrLZ by ERK1/2 promotes tumor cell survival *in vitro* and *in vivo*.

**IL-6 protects PrLZ from degradation through activating ERK1/2**

Interleukin (IL)-6 is a pro-inflammatory cytokine that has been identified as a key mediator in promotion of PCa growth, PCa progression to the castration-resistant state, promotion of cancer metastasis and resistance to chemotherapy[29]. Interestingly, modulation of ERK1/2 MAPKs signaling pathways is considered as one of the key molecular mechanisms for IL-6-mediated progression of PCa[30]. Previous research reported that IL-6 particularly induced PrLZ expression, whereas the expression of other TPD52 isoforms was not significantly affected[31]. We confirmed that IL-6 increased PrLZ protein levels and triggered the activation of ERK1/2 (Fig. 6a). Interestingly, no significant change of PrLZ S40A mutant and TPD52 protein levels were observed upon IL-6 treatment (Fig. S6a-d). It was thus reasonable to explore the effects of IL-6 on SPOP-mediated degradation of PrLZ. As shown in Figure 6b, SPOP-mediated degradation of PrLZ was abolished by IL-6 treatment. Additionally, ERK1/2 inhibitor SCH772984 attenuated IL-6-mediated upregulation of PrLZ (Fig. 6c). Furthermore, IL-6 weakened the interaction between SPOP and PrLZ, and SCH772984 treatment restored their interaction (Fig. 6d). Consequently, compared with DMSO treatment group, IL-6 conferred PrLZ the ability to resist ERK1/2-SPOP pathway-mediated ubiquitination and subsequent degradation (Fig. 6e-g). Importantly, neither IL-6 nor SCH772984 treatment had effects on PrLZ S40A or S40D expression and degradation (Fig. 6h and S6e-g).

We then chose EGF, another important upstream signal to activate ERK1/2[32], to determine whether other upstream molecules can be connected to the phosphorylation modification and protein degradation of PrLZ. We found the elevation of PrLZ protein level (Fig. S6h) and decreased degradation (Fig. S6i and S6j) of PrLZ upon EGF treatment. Additionally, we detected an increase in the phosphorylation of PrLZ WT instead of PrLZ S40D mutant upon IL-6 or EGF treatment (Fig. S6k and S6l). Importantly, similar *in vitro* ubiquitination level of BRD4 was observed upon incubation with IL-6 or ERK1, indicating that IL-6 or ERK1 might not directly regulate the activity of SPOP (Fig. S6m).

Functionally, we found that SCH772984 attenuated IL-6-induced C4-2 cell proliferation *in vitro* (Fig. S7a-c) and *in vivo* (Fig. S7d-f). We next examined the effects of PrLZ S40A mutation on cell proliferation and tumor growth under IL-6 and SCH772984 treatment. As shown in Fig. 6i-m, IL-6 treatment resulted in an increase of growth in cells and tumors expressing PrLZ WT, which could be abolished by SCH772984 treatment. However, we failed to observe the effects of IL-6 and SCH772984 in cells expressing PrLZ S40A mutant *in vitro* and *in vivo*.

Overall, the findings reveal that IL-6-induced upregulation of PrLZ in PCa partially depends on activation of ERK1/2, eventually leading to resistant to SPOP-mediated degradation.**Discussion**

In this study, we identified PrLZ as a novel substrate of Cullin 3/SPOP. More importantly, cancer-associated mutations clustered in the MATH domain of SPOP impaired the interaction between SPOP and PrLZ, thereby leading to the accumulation of PrLZ protein in PCa. Phosphorylation of PrLZ at Ser40 by ERK, an oncogenic isomerase previously demonstrated[33, 34], inhibited the SPOP- PrLZ interaction and reduced the ubiquitination of PrLZ. Our results deepen our understanding of the tumor suppressor function of SPOP and provide new insights into regulation of PCa oncogene PrLZ by SPOP-mediated ubiquitination and degradation.

ERK1/2, which belongs to the mitogen-activated protein kinase family, plays vital role in signal transduction and cancer progression[35]. ERK1/2 catalyzes the phosphorylation of substrates containing a PXS/TP sequence[36, 37]. ERK1/2 has gradually emerged as a potential therapeutic target for breast cancer, colorectal cancer, melanoma, pancreatic cancer and PCa[38]. ERK1/2 inhibitor such as SCH772984 has been proven to inhibit PCa cell proliferation[39, 40]. Here, our results identified ERK1/2 as the upstream kinase responsible for SPOP-mediated PrLZ poly-ubiquitination and degradation to favor PCa tumorigenesis. Accordingly, SCH772984 can inhibit PCa progression largely by promoting the SPOP-mediated degradation of PrLZ. Thus, our study authenticates the possibility of applying ERK1/2 inhibitors as novel therapeutic strategy for PCa, thereby providing a promising way to eliminate the tumor.

IL-6 is found to be elevated in a majority of cancers and involved in the malignant progression of cancers [41, 42, 43]. IL-6 could trigger ERK signaling and resulted in the promoting proliferation in PCa cells[44, 45]. It was reported that IL-6 induced a significant upregulation of PrLZ in PCa LNCaP cells and PrLZ overexpression enhanced the IL-6-mediated differentiation of LNCaP cells into a neuroendocrine-like phenotype, although the related molecular mechanism for PrLZ upregulation were unknown. In this study, we confirmed that IL-6/ERK signaling promoted phosphorylation and upregulation of PrLZ through SPOP-dependent regulation of PrLZ stability. On the basis of published *in vitro* and *in vivo* studies, one could conclude that options for targeting IL-6 signaling should be explored. In this context, the anti-IL-6 antibody siltuximab (CNTO 328) has been demonstrated to inhibit growth of PCa *in vitro* and *in vivo*. However, clinically, application of anti-IL-6 antibody did not improve survival of patients with castration therapy-resistant PCa[46, 47]. A difficulty with anti-IL-6 antibody monotherapy is the fact that multiple signaling pathways are deregulated at this stage. Thus a more detailed classification for prostate tumor microenvironment and validation of downstream molecules may be helpful in order to characterize the role of IL-6 in regulating PCa progression. Based on the specificity of PrLZ in PCa, an individualized approach is needed to identify PCa patients who will benefit from anti-IL-6 therapy in combination with PrLZ or ERK1/2 targeted therapy.

Structurally, the SPOP protein can selectively bind to specific substrates by recognizing their SBC motif (Φ-Π-S-S/T-S/T). Interestingly, we didn’t find the canonical SBC motif in the N-terminal region of PrLZ. Our data determined that amino acid 30-42 of PrLZ was responsible for the binding of PrLZ to SPOP and consequent SPOP-mediated degradation. Similar to some SPOP substrates which don’t have classic SBC motif[17], we found that PrLZ had a SBC-like motif (Φ-Π-S-X-S/T) in its 30-42 aa (^38^PGSPT^42^). We thus analyzed whether there were any pathologic mutations at amino acid 30-42 of PrLZ in cancers by analyzing TCGA and COSMIC databases (<https://cancer.sanger.ac.uk/cosmic/>). Although we have further identified that PrLZ Ser40 mutant nearly abolished SPOP-mediated degradation, we failed to find the pathologic Ser40 mutation of PrLZ based on databases. Within this context the question arises, are there any potential pathologic mutations located in amino acid 30-42 of PrLZ from patients with PCa? Will cancer-associated mutation of PrLZ impair its interaction with SPOP and evade SPOP-mediated degradation? Clearly, further in-depth studies are needed to address those questions.

In summary, aberrant accumulation of the PrLZ oncoprotein due to clinical SPOP mutation or abnormal activation of ERK1/2 in PCa could be the underlying molecular mechanisms driving tumor progression and resulting in poor survival of PCa patients (Fig. 7). To this end, our results verified ERK1/2 inhibitor as a novel therapeutic strategies *via* reducing PrLZ abundance based on the genetic status of SPOP mutation for PCa patients.

**Materials and Methods**

**Antibodies, Reagents and Plasmids.**

Antibodies green fluorescent protein (GFP) (6556), p27 (32034), Androgen Receptor (AR) (133273), Cullin 2 (166917), Cullin 4A (92554), Cullin 4B (67035), Cullin 5 (184177), SPOP (137537), ERK1/2 (17942), LC3B (51520) and TPD52 (182578) antibodies were purchased from Abcam. Antibodies Cullin 3 (2759), Cullin 1 (4995), Flag-tag (14793), HA-tag (3724), Myc-tag (2276), GST-tag (2624), Phospho-ERK1/2 (Thr202/Tyr204) (9101) and Vinculin (4650) were purchased from Cell Signaling Technology. Rabbit polyclonal PrLZ antibody was offered from Professor Ruoxiang Wang (Department of Medicine, Cedars-Sinai Medical Center, Los Angeles, CA, USA). pcDNA3-myc-Cullin 1, pcDNA3-myc-Cullin 2, pcDNA3-myc-Cullin 3, pcDNA3-myc-Cullin 4A, pcDNA3-myc-Cullin 4B, pcDNA3-myc-Cullin 5, pcDNA3-myc-ERK2, pcDNA3-Flag-SPOP, pcDNA3-Flag-PrLZ, pcDNA3-HA-SPOP, pcDNA3-HA-PrLZ, pCMV-GST-SPOP, pCMV-GST-PrLZ 1-46, pCMV-GST-PrLZ 1-46 S40A, pGEX-4T-1-GST-PrLZ, and pGEX-4T-1-GST-PrLZ S40A plasmids were constructed according to standard protocols by ourselves. KLHL2, KLHL3, PLZF, KLHL22, KLHL37, PrLZ delta 30-42, PrLZ S30A, PrLZ S32A, PrLZ S35A, PrLZ S36A, PrLZ S40A, PrLZ S40D, PrLZ T42A, TPD52, SPOP delta MATH and SPOP delta BTB were amplified and cloned into the pcDNA3-Flag vector. SPOP F102C, SPOP W131G, SPOP F133L, PrLZ 46-224, PrLZ 1-192, PrLZ 1-143, PrLZ 1-133, AMPK, ERK1, ERK2, GSK3β, CDK1, CDK2, CDK4 were amplified and cloned into the pcDNA3-HA or pLenti-HA vector. pCMV-8 x His-Ub (107392) was purchased from Addgene. pLKO-shCullin 1, pLKO-shCullin 2, pLKO-shCullin 3, pLKO-shCullin 4A, pLKO-shCullin 4B, pLKO-shCullin 5 and pLKO-shSPOP were purchased from Sigma-Aldrich. Oligonucleotide sequences for cloning shRNA constructs were as follows: shERK1 (5’-GATCCCCATGTCATAGGCATCCGAGATTCAAGAGATCTCGGATGCCTATGACATTTTTTGGAAA-3’) and shERK2 (5’-GATCCCCGACCGGATGTTAACCTTTATTCAAGAGATAAAGGTTAACATCCGGTCTTTTTGGAAA-3’).

siControl (sense, 5’-UUCUCCGAACGUGUCACGUTT-3’) and siSPOP (sense, 5’-AGAUCAAGGUAGUGAAAUUUU-3’ and 5’-GGUGAAGAGGGAACAGAAAUU-3’) were constructed by GE Dharmacon (GE Healthcare Ltd, Little Chalfont, UK). Different mutants were generated by site-directed mutagenesis PCR reaction using platinum PWO SuperYield DNA polymerase (Roche, Basel, Switzerland) according to the product manual.

**Cell culture, transfection and establishment of stable clone cells.**

Human PCa PC-3, C4-2 and 22Rv1 cell lines were purchased from the American Type Culture Collection (ATCC; Manassas, VA, USA) and cultured in RPMI-1640 medium containing 10% fetal bovine serum (FBS). In addition, the culture medium was supplemented with 100 U/mL penicillin and 0.1 mg/ml streptomycin (Gibco; Thermo Fisher Scientific, Inc.). The HEK293FT and HEK293T cell line was obtained from Professor Chawnshang Chang (Department of Urology, University of Rochester, Rochester, NY 14642, USA) and maintained in Dulbecco’s modified Eagle’s medium (DMEM) supplemented with 10% FBS. All established cell lines were cultivated for less than 6 months and were tested for mycoplasma every month.

Lipofectamine 2000 transfection reagent (Life Technologies) was applied to plasmid transfection in accordance with the manufacturer’s protocol. Various cell lines were infected with lentiviral and retroviral cDNA expressing viruses, which were packaged in HEK293FT cells. The SPOP-shRNA packaged into the lentiviral vector was transfect into PCa cells. Hygromycin B (200 μg/mL) was used in screening SPOP-shRNA cells. Two weeks later, PrLZ-shRNA packaged into the lentiviral vector was then transfect into PCa cells with the same lentiviral titer following by puromycin selection (1 μg/mL).

**Immunoprecipitation assay and immunoblot analysis**

Cells were lysed with EBC lysis buffer [50 mM of Tris-HCl (pH 7.4), 120 mM of NaCl, 5 mM of EDTA, 0.5% of NP-40] containing protease inhibitors (Sigma-Aldrich; Merck KGaA) and phosphatase inhibitors (Sigma-Aldrich; Merck KGaA). Beckman Coulter DU-800 spectrophotometer was applied in detecting the protein concentrations using the Bio-Rad protein assay reagent (Bio-Rad Laboratories, CA). The proteins were incubated with the monoclonal anti-FLAG or anti-HA antibody-conjugated M2 agarose beads (Sigma-Aldrich, A2095 and A2220) with gentle rocking at 4°C for 4 hours. Subsequently, cell lysates were washed with EBC buffer and the proteins were extracted from the beads by boiling at 95°C for 5 min. For immunoprecipitation assays, the proteins were seperated by sodium dodecyl sulfate-polyacrylamide gel electrophoresis (SDS-PAGE) gels. Then they were transferred to polyvinylidene fluoride (PVDF) membranes and incubated with specific primary antibodies at 4°C overnight. After incubating with HRP-conjugated secondary antibody for 1 h at room temperature, the protein immunoreactive signals were tested by ECL detection system (Thermo Fisher Scientific, Rochester, NY) or ECL chemiluminescence system (Santa Cruz Biotechnology). All the immunoblotting assays were repeated at least three times with similar results. For semi-quantitative analysis, immunoblot bands were analyzed using ImageJ software (NIH). All of the full and uncropped western blots are uploaded as Figure S8-S11.

**Total RNA extraction and real-time RT-PCR analysis.**

Total RNA of PCa cells was extracted with TRIzol and reversed by using a Reverse Transcription Reaction Kit (MBI Fermentas, St. Leon-Rot, Germany). Then cDNA was amplified using specific primers. Primer sequences were listed as follows: PrLZ (forward primer, 5’-GAGATGGACTTATATGAGGACTAC-3’; reverse primer, 5’-TTGCTGCTAACACTTGAGAC-3’) and β-actin (forward primer, 5’-TAATCTTCGCCTTAATACTT-3’; reverse primer, 5’-TAATCTTCGCCTTAATACTT-3’). Relative changes in gene expression were normalized against β-actin.

**Colony formation assay**

PCa cells were seeded into a 6-well plate (1.0×10^3^ cells/well) and cultured with RPMI-1640 medium supplemented with 10% FBS for 1 week. Then the cells were washed with phosphate-buffered saline and fixed with 4% paraformaldehyde for 15 min. The cells were then stained with crystal violet solution for 15 min. The staining solution was slowly washed away with running water, and then photographed with inverted microscope. All the colony formation assays were repeated at least three times.

***In vitro* ERK1 kinase assays**

Briefly, pGEX-4T-1 vector, pGEX-4T-1-GST-PrLZ and pGEX-4T-1-GST-PrLZ S40A were expressed in BL21 *Escherichia coli* and purified from transfected bacterial using Glutathione Sepharose 4B (GE Healthcare) according to the manufacturer’s instructions. For the *in vitro* kinase assay, recombinant ERK1 (R&D Systems) was incubated with 1 μg of purified proteins. Kinase assays were performed in a final volume of 30 μl of a kinase buffer: 50 mM HEPES (pH 7.5), 10 mM MgCl_2_, 1 mM DTT, 1 mM EGTA, 0.1 mM NaF, containing 10 μM ATP and 0.4 mCi [^32^P]γATP (Perkin Elmer). The proteins were denatured after incubating at 30°C for 60 min and then subjected to SDS-PAGE. Later on, the proteins were transferred to nitrocellulose membranes and exposed to X-ray films.

**GST pull-down assay**

In brief, pGEX-4T-1 vector (Addgene, 129567) and pGEX-4T-1-GST-PrLZ were expressed in BL21 Escherichia coli and purified from transfected bacterial using Glutathione Sepharose 4B beads (GE Healthcare, 17075605). pcDNA3-HA-SPOP was expressed in 293T and purified from transfected cells using HA-tag beads (Sigma-Aldrich, A2220). For GST pull-down assays, purified GST-PrLZ and HA-SPOP or His-SPOP (Abcam, 105599) proteins were incubated for 3 h with gentle rocking at 4°C. Later, the cell lysates were washed twice with RIPA buffer and boiled at 95°C for 5 min. Finally, the proteins were separated by western blotting as described.

**PrLZ Binding Assays**

GST-PrLZ WT and S40A proteins were isolated from BL21 Escherichia coli. Where indicated, the GST-PrLZ WT and S40A proteins were incubated with recombinant ERK1 (R&D Systems) in the presence of ATP for 60 min prior to the binding assays. For binding assay, purified proteins and ^35^S-labeled SPOP protein were incubated with Glutathione Sepharose 4B beads for 3 h with gentle rocking at 4°C. Later, the lysates were washed twice and bound proteins were eluted with SDS sample buffer, resolved by gel electrophoresis, and visualized by direct autoradiography.

***In Vivo* Ubiquitination Assays**

Cells were transfected with His-ubiquitin and indicated constructs for 42 hours and then treated with 20 mM MG132 for 6 hours. Subsequently, cells were lysed with buffer A (6 M guanidine-HCl, 0.1 M Na_2_HPO_4_/NaH_2_PO_4_, and 10 mM imidazole [pH 8.0]) and sonicated for 15 seconds. After incubating with nickel-nitrilotriacetic acid (Ni-NTA) beads (QIAGEN) for 3 hours at room temperature, the proteins were washed twice with buffer A, twice with buffer A/TI (1 volume buffer A and 3 volumes buffer TI), and one time with buffer TI (25 mM Tris-HCl and 20 mM imidazole [pH 6.8]). The pull-down proteins were denatured by boiling at 95°C for 5 min and seperated by SDS-PAGE for immunoblotting.

***In Vitro* Ubiquitination Assays**

For *in vitro* ubiquitination assays, SPOP protein with GST tag and BRD4-N (amino acids 1–500) protein with His tag were purified from BL21 Escherichia coli. To obtain the NEDDylated CUL3/RBX1, NEDD8 was first incubated with APP-BP1/Uba3, His-UBE2M enzymes at 30°C for 2 h in the presence of ATP, and followed by incubating with DCNL2 and CUL3/RBX1 at 4°C for 2 h. Then the NEDDylated CUL3/RBX1, GST-SPOP, Ub, E1, E2 (UbcH5a and UbcH5b) and His-BRD4-N (amino acids 1–500) were incubated with ATP (0.6 μl, 100 mM), ubiquitin aldehyde (1.5 μl, 20 μM), 3 μl 10 × ubiquitin reaction buffer (500 mM Tris-HCl (pH7.5), 50 mM KCl, 50 mM NaF, 50 mM MgCl2 and 5 mM DTT), 3 μl 10 × energy regeneration mix (200 mM creatine phosphate and 2 μg/μl creatine phosphokinase), 3 μl 10 × protease inhibitor cocktail at 30°C for 2 h, followed by western blot analysis. The Ub, E1, E2 and CUL3/RBX1 were purchased from UBIQUIGENT.

**Mass spectrometry analysis**

Cells were transfected with Flag-PrLZ, His-Ub and HA-SPOP. After 48 h, the cells were lysed with IP buffer and the transfected PrLZ was immunoprecipitated with Flag-agarose beads (Sigma-Aldrich, A2220). Gels were destained in 50 mM NH4HCO3 in 50% acetonitrile (v/v) and dehydrated with 100 μl of 100% acetonitrile for 5 min. After removing the liquid, the gels were rehydrated in 10 mM dithiothreitol at 56 °C for 1 hour. Gels were again dehydrated in 100% acetonitrile and rehydrated with 55 mM iodoacetamide for 45 min at room temperature in the dark. After washing with 50 mM NH4HCO3 and dehydrating with 100% acetonitrile, the gels were rehydrated with 10 ng/μl trypsin (Promega) and digested with trypsin at 37 °C overnight. Peptide mixtures were extracted with 50% acetonitrile/5% formic acid and 100% acetonitrile, followed by drying to completion and resuspending in 2% acetonitrile/0.1% formic acid. The tryptic peptides were dissolved in solvent A (0.1% formic acid), directly loaded onto a home-made reversed-phase analytical column (15-cm length, 75 μm i.d.). The gradient was comprised of an increase from 6% to 23% solvent B (0.1% formic acid in 98% acetonitrile) over 16 min, 23% to 35% in 8 min and climbing to 80% in 3 min then holding at 80% for the last 3 min, all at a constant flow rate of 400 nl/min on an EASY-nLC 1000 UPLC system. The peptides were subjected to NSI source followed by tandem mass spectrometry (MS/MS) in Q ExactiveTM Plus (Thermo Fisher Scientific) coupled online to the UPLC and the electrospray voltage applied was set to 2.0 kV. The m/z scan range was 100 to 1900 for full scan, and intact peptides were detected in the Orbitrap at a resolution of 70,000. Peptides were then selected for MS/MS using NCE setting as 28 and the fragments were detected in the Orbitrap at a resolution of 17,500. A data-dependent procedure that alternated between one MS scan followed by 20 MS/MS scans with 15.0s dynamic exclusion. The resulting MS/MS data were processed using Proteome Discoverer 1.3. Trypsin/P was specified as cleavage enzyme allowing up to 2 missing cleavages. Mass error was set to 10 ppm for precursor ions and 0.02 Da for fragment ions. Peptide confidence was set at high, and peptide ion score was set > 20. Ubiquitination sites were identified by allowing a dynamic modification of 114.1 Da to lysine residue. Phosphorylation sites were identified by allowing a dynamic modification of 80.0 Da to serine/threonine residue. The identified ubiquitinated or phosphorylated peptides were further examined by manually inspection to confirm the correct peptide sequence and the correct sites.

**Immunohistochemistry (IHC) for human PCa specimens**

The 128 PCa specimens were obtained from Shanghai Changhai Hospital (Shanghai, China) and the usage of these specimens was approved by the Institute Review Board of Shanghai Changhai Hospital. Detailed descriptions of patients’ information including SPOP mutation type, age, PSA level, Gleason score and tumor stage were supplied as a supplemental Table 1. For IHC in brief, specimens were deparaffinized and incubated with primary antibody against PrLZ or p-ERK1/2. After incubating with horseradish peroxidase-conjugated secondary antibody incubation for 1 hour at room temperature, the specimens were detected by using diaminobenzidine (DAB) and were finally observed under a microscope (Olympus Optical Co, Tokyo, Japan). We performed IHC staining of p-ERK1/2 and PrLZ expression in 128 PCa patients. In each patient, we used consecutive slides and analyzed the protein levels of p-ERK1/2 and PrLZ. The intensity of the IHC stain were scored as strong (3), intermediate (2), weak (1) and negative (0).

**PCa xenograft animal model**

Male BALB/c nude mice were purchased from the Laboratory Animal Center of Xi’an Jiaotong University. The administration of the animals was approved by the Institutional Animal Care and Use Committee of Xi’an Jiaotong University. Briefly, different groups of PCa cells were respectively resuspended with serum-free medium containing matrigel (Sigma-Aldrich; Merck KGaA), and 2.0×10^6^ cells were subcutaneously injected into right hind flanks of four weeks male BALB/c nude mice (n=6 per group). The tumor sizes were measured every three days by caliper after implantation for 1 week. For SCH772984 treatment group, xenografted mice were randomized and intraperitoneally injected with SCH772984 (50mg/kg, daily) when the tumor volume reached 100-150 mm^3^. For IL-6 treatment group, xenografted mice were intraperitoneally injected with IL-6 (100 ng per mouse, three times per week) and were administered 1 day before tumor implantation. The tumor volume was calculated by reference to the following formula: volume (mm^3^) = 1/2 × (length) × (width)^2^. Thirty days later, the mice were sacrificed. Tumors were then weighted and prepared for subsequent experiments.

**Statistical analysis.**

All data are presented as the mean ± standard deviation (SD). All assays were repeated at least three times. The difference between various groups were analyzed by one-way ANOVA. Student’s *t* test was used for the comparison between only two groups. Mann-Whitney test was used for the IHC analysis. *P*<0.05 was served as the criterion to represent the significant difference.

**Data availability**

The data supporting the present study are available from the corresponding author upon reasonable request.

**Funding**

This work was supported by grants from the National Natural Science Foundation of China (NO. 81925028, 82073304, 82002694 and 81773206).

**Author contributions**

Y.Z.F., T.H., W.C.D. and Y.S.Z. have contributed equally to this work. J.Z. and L.L. designed and supervised all experiments and contributed to the manuscript preparation. Y.Z.F., T.H., W.C.D., Y.S.Z., B.L., Y.W., Z.X.W., Y.G. and J.Z. performed the experiments, analyzed the data and contributed to the manuscript preparation. Y.Z.F., T.H. and Y.S.Z. collected animal samples and participated in the animal experiments. Y.Z.F., J.Z., Y.G. and L.L., wrote the manuscript.

**Conflict of interest**

The authors have declared that no competing interest exists.

**Ethical approval**

All animal experiments conducted were compliant with Ethics Committee of Xi’an JiaoTong University.

**References**

1. Rawla P. Epidemiology of Prostate Cancer. *World J Oncol* 2019, **10**(2)**:** 63-89.

2. Wang R, Xu J, Saramäki O, Visakorpi T, Sutherland WM, Zhou J*, et al.* PrLZ, a novel prostate-specific and androgen-responsive gene of the TPD52 family, amplified in chromosome 8q21.1 and overexpressed in human prostate cancer. *Cancer research* 2004, **64**(5)**:** 1589-1594.

3. Li L, Zhang D, Zhang L, Zhu G, Sun Y, Wu K*, et al.* PrLZ expression is associated with the progression of prostate cancer LNCaP cells. *Mol Carcinog* 2009, **48**(5)**:** 432-440.

4. Zhang D, He D, Xue Y, Wang R, Wu K, Xie H*, et al.* PrLZ protects prostate cancer cells from apoptosis induced by androgen deprivation via the activation of Stat3/Bcl-2 pathway. *Cancer research* 2011, **71**(6)**:** 2193-2202.

5. Li L, Xie H, Liang L, Gao Y, Zhang D, Fang L*, et al.* Increased PrLZ-mediated androgen receptor transactivation promotes prostate cancer growth at castration-resistant stage. *Carcinogenesis* 2013, **34**(2)**:** 257-267.

6. Zeng J, Liu W, Fan YZ, He DL, Li L. PrLZ increases prostate cancer docetaxel resistance by inhibiting LKB1/AMPK-mediated autophagy. *Theranostics* 2018, **8**(1)**:** 109-123.

7. Fan Y, Hou T, Gao Y, Dan W, Liu T, Liu B*, et al.* Acetylation-dependent regulation of TPD52 isoform 1 modulates chaperone-mediated autophagy in prostate cancer. *Autophagy* 2021**:** 1-15.

8. Wang Z, Song Y, Ye M, Dai X, Zhu X, Wei W. The diverse roles of SPOP in prostate cancer and kidney cancer. *Nat Rev Urol* 2020, **17**(6)**:** 339-350.

9. Liu D, Augello MA, Grbesa I, Prandi D, Liu Y, Shoag JE*, et al.* Tumor subtype defines distinct pathways of molecular and clinical progression in primary prostate cancer. *The Journal of clinical investigation* 2021, **131**(10).

10. Cheng J, Guo J, Wang Z, North BJ, Tao K, Dai X*, et al.* Functional analysis of Cullin 3 E3 ligases in tumorigenesis. *Biochim Biophys Acta Rev Cancer* 2018, **1869**(1)**:** 11-28.

11. An J, Wang C, Deng Y, Yu L, Huang H. Destruction of full-length androgen receptor by wild-type SPOP, but not prostate-cancer-associated mutants. *Cell Rep* 2014, **6**(4)**:** 657-669.

12. Dai X, Gan W, Li X, Wang S, Zhang W, Huang L*, et al.* Prostate cancer-associated SPOP mutations confer resistance to BET inhibitors through stabilization of BRD4. *Nat Med* 2017, **23**(9)**:** 1063-1071.

13. Zhang P, Wang D, Zhao Y, Ren S, Gao K, Ye Z*, et al.* Intrinsic BET inhibitor resistance in SPOP-mutated prostate cancer is mediated by BET protein stabilization and AKT-mTORC1 activation. *Nat Med* 2017, **23**(9)**:** 1055-1062.

14. Geng C, He B, Xu L, Barbieri CE, Eedunuri VK, Chew SA*, et al.* Prostate cancer-associated mutations in speckle-type POZ protein (SPOP) regulate steroid receptor coactivator 3 protein turnover. *Proc Natl Acad Sci U S A* 2013, **110**(17)**:** 6997-7002.

15. Theurillat JP, Udeshi ND, Errington WJ, Svinkina T, Baca SC, Pop M*, et al.* Prostate cancer. Ubiquitylome analysis identifies dysregulation of effector substrates in SPOP-mutant prostate cancer. *Science* 2014, **346**(6205)**:** 85-89.

16. Groner AC, Cato L, de Tribolet-Hardy J, Bernasocchi T, Janouskova H, Melchers D*, et al.* TRIM24 Is an Oncogenic Transcriptional Activator in Prostate Cancer. *Cancer cell* 2016, **29**(6)**:** 846-858.

17. Zhang J, Bu X, Wang H, Zhu Y, Geng Y, Nihira NT*, et al.* Cyclin D-CDK4 kinase destabilizes PD-L1 via cullin 3-SPOP to control cancer immune surveillance. *Nature* 2018, **553**(7686)**:** 91-95.

18. Tang Z, Pilié PG, Geng C, Manyam GC, Yang G, Park S*, et al.* ATR Inhibition Induces CDK1-SPOP Signaling and Enhances Anti-PD-L1 Cytotoxicity in Prostate Cancer. *Clinical cancer research : an official journal of the American Association for Cancer Research* 2021, **27**(17)**:** 4898-4909.

19. Shi L, Yan Y, He Y, Yan B, Pan Y, Orme JJ*, et al.* Mutated SPOP E3 Ligase Promotes 17βHSD4 Protein Degradation to Drive Androgenesis and Prostate Cancer Progression. *Cancer research* 2021, **81**(13)**:** 3593-3606.

20. Jin X, Qing S, Li Q, Zhuang H, Shen L, Li J*, et al.* Prostate cancer-associated SPOP mutations lead to genomic instability through disruption of the SPOP-HIPK2 axis. *Nucleic acids research* 2021, **49**(12)**:** 6788-6803.

21. Bernasocchi T, El Tekle G, Bolis M, Mutti A, Vallerga A, Brandt LP*, et al.* Dual functions of SPOP and ERG dictate androgen therapy responses in prostate cancer. *Nature communications* 2021, **12**(1)**:** 734.

22. Gan W, Dai X, Lunardi A, Li Z, Inuzuka H, Liu P*, et al.* SPOP Promotes Ubiquitination and Degradation of the ERG Oncoprotein to Suppress Prostate Cancer Progression. *Mol Cell* 2015, **59**(6)**:** 917-930.

23. Li G, Ci W, Karmakar S, Chen K, Dhar R, Fan Z*, et al.* SPOP promotes tumorigenesis by acting as a key regulatory hub in kidney cancer. *Cancer cell* 2014, **25**(4)**:** 455-468.

24. Wang L, Lin M, Chu M, Liu Y, Ma J, He Y*, et al.* SPOP promotes ubiquitination and degradation of LATS1 to enhance kidney cancer progression. *EBioMedicine* 2020, **56:** 102795.

25. Guo ZQ, Zheng T, Chen B, Luo C, Ouyang S, Gong S*, et al.* Small-Molecule Targeting of E3 Ligase Adaptor SPOP in Kidney Cancer. *Cancer cell* 2016, **30**(3)**:** 474-484.

26. Ding M, Lu X, Wang C, Zhao Q, Ge J, Xia Q*, et al.* The E2F1-miR-520/372/373-SPOP Axis Modulates Progression of Renal Carcinoma. *Cancer research* 2018, **78**(24)**:** 6771-6784.

27. Wang X, Jin J, Wan F, Zhao L, Chu H, Chen C*, et al.* AMPK Promotes SPOP-Mediated NANOG Degradation to Regulate Prostate Cancer Cell Stemness. *Dev Cell* 2019, **48**(3)**:** 345-360.e347.

28. Song Y, Xu Y, Pan C, Yan L, Wang ZW, Zhu X. The emerging role of SPOP protein in tumorigenesis and cancer therapy. *Mol Cancer* 2020, **19**(1)**:** 2.

29. Culig Z, Puhr M. Interleukin-6 and prostate cancer: Current developments and unsolved questions. *Molecular and cellular endocrinology* 2018, **462**(Pt A)**:** 25-30.

30. Nguyen DP, Li J, Tewari AK. Inflammation and prostate cancer: the role of interleukin 6 (IL-6). *BJU international* 2014, **113**(6)**:** 986-992.

31. Moritz T, Venz S, Junker H, Kreuz S, Walther R, Zimmermann U. Isoform 1 of TPD52 (PC-1) promotes neuroendocrine transdifferentiation in prostate cancer cells. *Tumour biology : the journal of the International Society for Oncodevelopmental Biology and Medicine* 2016, **37**(8)**:** 10435-10446.

32. Miyamoto Y, Suyama K, Baba H. Recent Advances in Targeting the EGFR Signaling Pathway for the Treatment of Metastatic Colorectal Cancer. *International journal of molecular sciences* 2017, **18**(4).

33. Roskoski R, Jr. Targeting ERK1/2 protein-serine/threonine kinases in human cancers. *Pharmacol Res* 2019, **142:** 151-168.

34. Kidger AM, Sipthorp J, Cook SJ. ERK1/2 inhibitors: New weapons to inhibit the RAS-regulated RAF-MEK1/2-ERK1/2 pathway. *Pharmacol Ther* 2018, **187:** 45-60.

35. Sun Y, Liu WZ, Liu T, Feng X, Yang N, Zhou HF. Signaling pathway of MAPK/ERK in cell proliferation, differentiation, migration, senescence and apoptosis. *J Recept Signal Transduct Res* 2015, **35**(6)**:** 600-604.

36. Carlson SM, Chouinard CR, Labadorf A, Lam CJ, Schmelzle K, Fraenkel E*, et al.* Large-scale discovery of ERK2 substrates identifies ERK-mediated transcriptional regulation by ETV3. *Sci Signal* 2011, **4**(196)**:** rs11.

37. Lefloch R, Pouysségur J, Lenormand P. Total ERK1/2 activity regulates cell proliferation. *Cell Cycle* 2009, **8**(5)**:** 705-711.

38. Caunt CJ, Sale MJ, Smith PD, Cook SJ. MEK1 and MEK2 inhibitors and cancer therapy: the long and winding road. *Nat Rev Cancer* 2015, **15**(10)**:** 577-592.

39. Broutier L, Mastrogiovanni G, Verstegen MM, Francies HE, Gavarró LM, Bradshaw CR*, et al.* Human primary liver cancer-derived organoid cultures for disease modeling and drug screening. *Nat Med* 2017, **23**(12)**:** 1424-1435.

40. Li J, Wang R, Hu X, Gao Y, Wang Z, Li J*, et al.* Activated MEK/ERK Pathway Drives Widespread and Coordinated Overexpression of UHRF1 and DNMT1 in Cancer cells. *Sci Rep* 2019, **9**(1)**:** 907.

41. Johnson DE, O'Keefe RA, Grandis JR. Targeting the IL-6/JAK/STAT3 signalling axis in cancer. *Nat Rev Clin Oncol* 2018, **15**(4)**:** 234-248.

42. Kumari N, Dwarakanath BS, Das A, Bhatt AN. Role of interleukin-6 in cancer progression and therapeutic resistance. *Tumour biology : the journal of the International Society for Oncodevelopmental Biology and Medicine* 2016, **37**(9)**:** 11553-11572.

43. Kampan NC, Xiang SD, McNally OM, Stephens AN, Quinn MA, Plebanski M. Immunotherapeutic Interleukin-6 or Interleukin-6 Receptor Blockade in Cancer: Challenges and Opportunities. *Curr Med Chem* 2018, **25**(36)**:** 4785-4806.

44. Lin DL, Whitney MC, Yao Z, Keller ET. Interleukin-6 induces androgen responsiveness in prostate cancer cells through up-regulation of androgen receptor expression. *Clinical cancer research : an official journal of the American Association for Cancer Research* 2001, **7**(6)**:** 1773-1781.

45. Yang L, Wang L, Lin HK, Kan PY, Xie S, Tsai MY*, et al.* Interleukin-6 differentially regulates androgen receptor transactivation via PI3K-Akt, STAT3, and MAPK, three distinct signal pathways in prostate cancer cells. *Biochem Biophys Res Commun* 2003, **305**(3)**:** 462-469.

46. Dorff TB, Goldman B, Pinski JK, Mack PC, Lara PN, Jr., Van Veldhuizen PJ, Jr.*, et al.* Clinical and correlative results of SWOG S0354: a phase II trial of CNTO328 (siltuximab), a monoclonal antibody against interleukin-6, in chemotherapy-pretreated patients with castration-resistant prostate cancer. *Clinical cancer research : an official journal of the American Association for Cancer Research* 2010, **16**(11)**:** 3028-3034.

47. Fizazi K, De Bono JS, Flechon A, Heidenreich A, Voog E, Davis NB*, et al.* Randomised phase II study of siltuximab (CNTO 328), an anti-IL-6 monoclonal antibody, in combination with mitoxantrone/prednisone versus mitoxantrone/prednisone alone in metastatic castration-resistant prostate cancer. *European journal of cancer (Oxford, England : 1990)* 2012, **48**(1)**:** 85-93.

**Figure Legends:**

**Figure 1. Cullin 1 and Cullin 3 E3 ubiquitin ligases negatively regulate PrLZ protein stability. a** Immunoblot (IB) analysis of whole-cell lysates (WCL) derived from C4-2 cells treated with MG132 (5 and 10 μM) or MLN4924 (0.5 and 1 μM) for 12 hours. **b** IB analysis of WCL derived from 22Rv1 cells under similar treatment condition. **c** PrLZ binds with Cullin 1 and Cullin 3. IB analysis of WCL and anti-Myc immunoprecipitates (IPs) derived from 293T cells transfected with Flag-PrLZ and indicated Myc-tagged Cullins. EV, empty vector. **d** IB analysis of WCL and anti-Flag IPs derived from 293T cells transfected with Flag-PrLZ and Myc-Cullin 3. EV, empty vector. **e** IB analysis of WCL derived from C4-2 and 22Rv1 cells stably expressing shCullin 3 or shScr. Scr, Scramble. **f** IB analysis of WCL derived from C4-2 cells transfected with increasing transfection doses (0.5, 1.5 and 3 μg) of Myc-Cullin 3. EV, empty vector. **g** IB analysis of WCL derived from 293T cells transfected with Flag-PrLZ and increasing transfection doses (1.5 and 3 μg) of Myc-Cullin 3. EV, empty vector. **h** IB analysis of WCL and Ni-NTA pull-down products derived from PC-3 cells transfected with Flag-PrLZ, Myc-Cullin 3 and His-Ub. Where indicated, 20 μM MG132 was added for 6 hours before harvesting the cells. **i** Cullin 3 knockdown cells (shCullin 3) as well as parental C4-2 cells (shScr) were treated with 100 μg/ml cycloheximide (CHX) for the indicated time period before harvesting. Equal amounts of WCL were immunoblotted with the indicated antibodies. **j** The PrLZ protein abundance in (**i**) was quantified by ImageJ and plotted as indicated. PrLZ bands were normalized to vinculin.

**Figure 2. SPOP specifically interacts with and promotes PrLZ poly-ubiquitination and degradation. a** PrLZ binds with SPOP. Immunoblot (IB) analysis of WCL and anti-HA immunoprecipitates (IPs) derived from 293T cells transfected with HA-PrLZ and Flag-tagged BTB domain-containing protein constructs. 30 hours post-transfection, cells were treated with 20 μM MG132 for 6 hours before harvesting. EV, empty vector. **b** IB analysis of WCL and anti-SPOP IPs derived from C4-2. Cells were treated with 20 μM MG132 for 6 hours before harvesting. **c** GST pull-down assay revealed the direct interaction between PrLZ and SPOP. The upper panel presents the result of IB by using the antibody against HA, and the lower coomassie blue staining showing the gels for purified proteins. **d** IB analysis of WCL derived from C4-2 cells with *SPOP* knockout by the CRISPR-Cas9 technology. Parental C4-2 cells are used as the control. **e** IB analysis of WCL derived from 22Rv1 cells with *SPOP* knockout by the CRISPR-Cas9 technology. Parental 22Rv1 cells are used as the control. **f** IB analysis of WCL derived from C4-2 cells transfected with increasing transfection doses (1 and 2 μg) of HA-SPOP. EV, empty vector. **g** SPOP knockout cells (sgSPOP) as well as parental C4-2 cells (Control) were treated with 100 μg/ml CHX for the indicated time period before harvesting. Equal amounts of WCL were immunoblotted with the indicated antibodies. **h** The PrLZ protein abundance in (**g**) was quantified by ImageJ and plotted as indicated. PrLZ bands were normalized to vinculin. **i** IB analysis of WCL and Ni-NTA pull-down products derived from PC-3 cells transfected with Flag-PrLZ, HA-SPOP, Myc-Cullin 3 and His-Ub. Where indicated, 20 μM MG132 was added for 6 hours before harvesting the cells. **j** IB analysis of WCL and Ni-NTA pull-down products derived from PC-3 cells transfected with Flag-PrLZ, HA-SPOP and K-only ubiquitin mutants. Where indicated, 20 μM MG132 was added for 6 hours before harvesting the cells. **k** IB analysis of WCL and Ni-NTA pull-down products derived from PC-3 cells transfected with Flag-PrLZ, HA-SPOP and the indicated ubiquitin KR (Lys to Arg) mutants. Where indicated, 20 μM MG132 was added for 6 hours before harvesting the cells. **l** IB analysis of WCL and Ni-NTA pull-down products derived from PC-3 cells transfected with Flag-tagged wild type (WT) and mutanted PrLZ, HA-SPOP and His-Ub. Where indicated, 20 μM MG132 was added for 6 hours before harvesting the cells. **m** The growth curve of C4-2 cells with knockdown of SPOP and/or PrLZ. Scr, Scramble. **P* < 0.05. **n-o** Colony formation assays and quantification of C4-2 cells with knockdown of SPOP and/or PrLZ. Scr, Scramble. Error bars represent SEs. **P* < 0.05. **p-q** Representative images and quantification of migrated C4-2 cells with knockdown of SPOP and/or PrLZ. Scr, Scramble. Error bars represent SEs. **P* < 0.05.

**Figure 3. PCa-associated SPOP Mutants fail to interact with and promote PrLZ poly-ubiquitination and degradation. a** A schematic diagram representing the SPOP structural domains and PCa-associated mutations for mapping the interaction domains with PrLZ. **b** Immunoblot (IB) analysis of WCL and anti-Flag immunoprecipitates (IPs) derived from 293T cells transfected with Flag-PrLZ, HA-SPOP WT, deletion of MATH domain-SPOP constructs and BTB domain-SPOP constructs. 30 hours post-transfection, cells were treated with 20 μM MG132 for 6 hours before harvesting. EV, empty vector. WT, wild type. **c** IB analysis of WCL derived from 293T cells transfected with Flag-PrLZ, HA-SPOP WT and deletion of MATH domain-SPOP constructs. EV, empty vector. WT, wild type. **d** IB analysis of WCL and anti-Flag IPs derived from 293T cells transfected with Flag-PrLZ, HA-SPOP WT and PCa-associated SPOP mutants. EV, empty vector. WT, wild type. **e** IB analysis of WCL derived from C4-2 cells stably expressing HA-tagged SPOP WT or PCa-associated SPOP mutants. EV, empty vector. WT, wild type. **f** IB analysis of WCL derived from 293T cells transfected with Flag-PrLZ, HA-SPOP WT and HA-SPOP F102C mutant. Where indicated, 100 μg/ml CHX was added for the indicated time period before harvesting. EV, empty vector. WT, wild type. **g** The PrLZ protein abundance in (**f**) was quantified by ImageJ and plotted as indicated. PrLZ bands were normalized to vinculin. **h** IB analysis of WCL and Ni-NTA pull-down products derived from PC-3 cells transfected with Flag-PrLZ, HA-SPOP WT, HA-SPOP F102C mutant and HA-SPOP W131G mutant and His-Ub. Where indicated, 20 μM MG132 was added for 6 hours before harvesting the cells. EV, empty vector. WT, wild type. **i-k** C4-2 cells stably expressing SPOP-F102C or SPOP-W131G mutants were transfected with shPrLZ or shScr and subcutaneously injected into nude mice to establish xenograft model. Statistical analysis of the tumor volumes which were measured every three days and plotted individually (**i**). Subcutaneous xenograft tumors formed from different groups were dissected (**j**). Statistical analysis of the weight of the dissected xenografts tumors (**k**). n = 6 mice per experimental group, the results indicate the mean ± S.D. ^*^*P*＜0.05. Scr, Scramble. **l** Representative images of primary PCa patient samples stained for PrLZ expression by immunohistochemistry. Scale bar, upper 200 μm, lower 100 μm. **m** Mann-Whitney test analysis of PrLZ expression in primary PCa patient samples harboring *SPOP*-WT or *SPOP*-mutations.

**Figure 4. SPOP promotes PrLZ ubiquitination and degradation through interaction with the distinctive N-terminal of PrLZ. a** A schematic diagram representing the PrLZ structural domains for mapping the interaction domains with SPOP. **b** Immunoblot (IB) analysis of WCL and anti-HA immunoprecipitates (IPs) derived from 293T cells transfected with GST-SPOP and indicated constructs of HA-PrLZ. 30 hours post-transfection, cells were treated with 20 μM MG132 for 6 hours before harvesting. EV, empty vector. WT, wild type. **c** IB analysis of WCL derived from 293T cells transfected with HA-PrLZ WT, HA-PrLZ animo acid (aa) 46-224 constructs and increasing transfection doses (1 and 3 μg) of Flag-SPOP. WT, wild type. **d** IB analysis of WCL and anti-Flag IPs derived from 293T cells transfected with HA-SPOP, Flag-PrLZ WT and deletion of aa 30-42 domain-PrLZ constructs. 30 hours post-transfection, cells were treated with 20 μM MG132 for 6 hours before harvesting. EV, empty vector. WT, wild type. **e** IB analysis of WCL derived from 293T cells transfected with HA-SPOP, Flag-PrLZ WT and deletion of aa 30-42 domain-PrLZ constructs. EV, empty vector. WT, wild type. **f** IB analysis of WCL and Ni-NTA pull-down products derived from PC-3 cells transfected with HA-SPOP, His-Ub, Flag-PrLZ WT and deletion of aa 30-42 domain-PrLZ constructs. Where indicated, 20 μM MG132 was added for 6 hours before harvesting the cells. EV, empty vector. WT, wild type. **g** IB analysis of WCL and anti-Flag IPs derived from 293T cells transfected with HA-SPOP and indicated mutation constructs of Flag-PrLZ. EV, empty vector. WT, wild type. **h** IB analysis of WCL and Ni-NTA pull-down products derived from PC-3 cells transfected with HA-SPOP, His-Ub, Flag-PrLZ WT and Flag-PrLZ S40A mutant. Where indicated, 20 μM MG132 was added for 6 hours before harvesting the cells. EV, empty vector. WT, wild type. **i** IB analysis of WCL derived from 293T cells transfected with HA-SPOP, Flag-PrLZ WT and Flag-PrLZ S40A mutant. Where indicated, 100 μg/ml CHX was added for the indicated time period before harvesting. WT, wild type. **j** The PrLZ protein abundance in (**i**) was quantified by ImageJ and plotted as indicated. PrLZ bands were normalized to vinculin. **k** IB analysis of WCL derived from 293T cells transfected with HA-SPOP and pull-down binding assay using biotinylated peptide.

**Figure 5. ERK1/2 mediated phosphorylation of PrLZ at Ser40 stabilizes PrLZ through disrupting its binding with SPOP. a** Post-translational modifications of PrLZ identified by mass spectrometry (MS). Flag-PrLZ protein derived from 293T cells were immunoprecipitated with anti-Flag antibody, separated by SDS–PAGE gel and subjected to in-gel digestion for MS. MS/MS spectrum of the PrLZ fragment from S30 to T42, m/z=672.312 (Z=2) was shown. **b** Immunoblot (IB) analysis of WCL and anti-Flag immunoprecipitates (IPs) derived from 293T cells transfected with HA-SPOP, Flag-PrLZ WT and Flag-PrLZ S40D mutant. 30 hours post-transfection, cells were treated with 20 μM MG132 for 6 hours before harvesting. EV, empty vector. WT, wild type. **c** IB analysis of WCL derived from 293T cells transfected with HA-SPOP, Flag-PrLZ WT and Flag-PrLZ S40D mutant. Where indicated, 100 μg/ml CHX was added for the indicated time period before harvesting. WT, wild type. **d** IB analysis of WCL derived from 293T cells transfected with GST-SPOP, Flag-PrLZ WT and indicated kinases constructs. EV, empty vector. **e** IB analysis of WCL and anti-HA IPs derived from 293T cells transfected with Flag-PrLZ, HA-ERK1 and HA-ERK2. 30 hours post-transfection, cells were treated with 20 μM MG132 for 6 hours before harvesting. EV, empty vector. **f** IB analysis of WCL derived from C4-2 cells treated with SCH772984 (1 and 3 μM) for 24 hours. **g** Sequence alignment of phosphorylation of PrLZ within the SPOP binding domain. **h** *In vitro* kinase assays showing that ERK1 phosphorylated recombinant PrLZ at Ser40. **i** ERK1-mediated phosphorylation of PrLZ hindered its interaction with SPOP *in vitro*. Autoradiograms showing recovery of ^35^S-labeled SPOP protein bound to the indicated GST-PrLZ fusion proteins (GST protein as a negative control). IN, input (5% as indicated). **j** IB analysis of WCL derived from C4-2 and 22Rv1 cells stably expressing shERK1/2 or shScr. Scr, Scramble. **k** IB analysis of WCL and anti-HA IPs derived from 293T cells transfected with Flag-PrLZ and HA-SPOP. 12 hours post-transfection, cells were treated with 3 μM SCH772984 for additional 24 hours before harvesting. Where indicated, 20 μM MG132 was added for 6 hours before harvesting the cells. **l** IB analysis of WCL derived from C4-2 cells treated with or without 3 μM SCH772984. Where indicated, 100 μg/ml CHX was added for the indicated time period before harvesting. WT, wild type. **m** The PrLZ protein abundance in (**l**) was quantified by ImageJ and plotted as indicated. PrLZ bands were normalized to vinculin. **n** IB analysis of WCL and Ni-NTA pull-down products derived from PC-3 cells transfected with Flag-PrLZ, HA-SPOP and His-Ub. 12 hours post-transfection, cells were treated with 3 μM SCH772984 for 24 hours before harvesting. Where indicated, 20 μM MG132 was added for 6 hours before harvesting the cells. **o-q** PC-3 cells stably expressing PrLZ-WT or PrLZ-S40A mutant were subcutaneously injected into nude mice with or without SCH772984 treatment (50mg/kg, daily). Statistical analysis of the tumor volumes which were measured every three days and plotted individually **(o)**. Subcutaneous xenograft tumors from different groups in PC-3 cells were dissected **(p)**. Statistical analysis of the weights of the dissected xenografts tumors **(q)**. n = 6 mice per experimental group, the results indicated the mean ± S.D. ^*^*P*＜0.05 and ^***^*P*＜0.001. NC, non-specific control. WT, wild type. SCH, SCH772984. **r** Representative images of PCa patient samples stained for PrLZ and p-ERK1/2 expression by immunohistochemistry. Scale bar, left 200 μm, right 100 μm. **s** Correlation analysis of PrLZ and p-ERK1/2 expression in PCa patient samples. **t** Mann-Whitney test analysis of PrLZ expression in p-ERK1/2 negative and positive PCa patient samples.

**Figure 6. IL-6 protects PrLZ from degradation through activating ERK1/2.**

**a** Immunoblot (IB) analysis of WCL derived from C4-2 and 22Rv1 cells treated with different concentration of IL-6 (10, 25, 50 and 100 ng/ml) for 48 hours. **b** IB analysis of WCL derived from C4-2 cells treated with IL-6 (50 ng/ml, 48 h) or/and transfection with HA-SPOP. **c** IB analysis of WCL derived from C4-2 cells treated with IL-6 (50 ng/ml, 48 h) or/and SCH772984 (3 μM). **d** IB analysis of WCL and anti-Flag immunoprecipitates (IPs) derived from 293T cells transfected with HA-SPOP and Flag-PrLZ. Cells were treated with IL-6 (50 ng/ml, 48 h) or/and SCH772984 (3 μM). 30 hours post-transfection, cells were treated with 20 μM MG132 for 6 hours before harvesting. **e** IB analysis of WCL and Ni-NTA pull-down products derived from PC-3 cells transfected with Flag-PrLZ, HA-SPOP and His-Ub. Cells were treated with IL-6 (50 ng/ml) or/and SCH772984 (3 μM). Where indicated, 20 μM MG132 was added for 6 hours before harvesting the cells. **f** IB analysis of WCL derived from C4-2 cells treated with IL-6 (50 ng/ml) or/and SCH772984 (3 μM). Where indicated, 100 μg/ml CHX was added for the indicated time period before harvesting. **g** The PrLZ protein abundance in (**f**) was quantified by ImageJ and plotted as indicated. PrLZ bands were normalized to vinculin. **h** IB analysis of WCL derived from C4-2 cells transfected with Flag-PrLZ WT and Flag-PrLZ S40A. Cells were treated with IL-6 (50 ng/ml) or/and SCH772984 (3 μM). **i-j** Colony formation assays and quantitative analysis of C4-2 cells transfected with Flag-PrLZ WT or Flag-PrLZ S40A in the presence or absence of IL-6 (50 ng/ml) or/and SCH772984 (3 μM) treatment. ***P* < 0.05. **k-m** C4-2 cells transfected with PrLZ WT or PrLZ S40A were subcutaneously injected into nude mice, which were treated with IL-6 (100 ng per mouse) or/and SCH772984 (SCH, 50mg/kg, daily). Statistical analysis of the tumor volumes which were measured every three days and plotted individually **(k)**. Subcutaneous xenograft tumors formed from different groups in C4-2 cells were dissected **(l)**. Statistical analysis of the weights of the dissected xenografts tumors **(m)**. n = 6 mice per experimental group, the results indicated the mean ± S.D. ^*^*P*＜0.05.

**Figure 7. Graphical summary of the proposed mechanism.**A schematic diagram showing the working model for phosphorylation-regulated PrLZ ubiquitination and degradation in modulating PCa progression.
