# Supplementary material for: ERK1/2 inhibits Cullin 3/SPOP-mediated PrLZ ubiquitination and degradation to modulate prostate cancer progression
Source: Cell Death Differ. 2022 Feb 22;29(8):1611–24. doi: 10.1038/s41418-022-00951-y (PMC9345960; doi:10.1038/s41418-022-00951-y)
Supplement: Supplementary file 4 — Change of authorship request form [file 41418_2022_951_MOESM4_ESM.pdf]

SPRINGER NATURE

Change of authorship request form - Journals (pre-acceptance)

Important information. Please read.

- This form should be used by authors to request any change in authorship (adding/deleting authors) including changes in corresponding authors. This form should not be used for name changes. Please fully complete all sections. Use black ink and block capitals and provide each author's full name with the given name first followed by the family name.
- By signing this declaration, all authors guarantee that the order of the authors are in accordance with their scientific contribution, if applicable as different conventions apply per discipline, and that only authors have been added who made a meaningful contribution to the work.
- Please note, in author collaborations where there is formal agreement for representing the collaboration, it is sufficient for the representative or legal guarantor (usually the corresponding author) to complete and sign the Authorship Change Form on behalf of all authors, **next to the added/removed author(s)**. **(Complete Section 3, followed by Section 6.)**
  In author collaborations where there is no formal agreement for representing the collaboration and **there are more than 10 authors**, one may sign for all, provided the signer appends correspondence that attests that each of the authors have agreed to the change **and the added/removed authors sign the form**. **(Complete Section 3, followed by Section 6.)**
- Please note, we cannot investigate or mediate any authorship disputes. If you are unable to obtain agreement from all authors (including those who you wish to be removed) you must refer the matter to your institution(s) for investigation. Please inform us if you need to do this.
- If you are not able to return a fully completed form within **30 days** of the date that it was sent to the author requesting the change, we may have to withdraw your manuscript. We cannot publish manuscripts where authorship has not been agreed by all authors (including those who have been removed).
- Incomplete forms will be rejected.
- Please return/upload this form, fully completed, to the Journals Editorial Office. The Journal and/or Publisher will consider the information you have provided to decide whether to approve the proposed change in authorship. We may decide to contact your institution for more information or undertake a further investigation, if appropriate, before making a final decision.

SPRINGER NATURE

Change of authorship request form - Journals (pre-acceptance)

Section 1: Please provide the current title of manuscript

Manuscript ID no.: CDD-21-1441R

Title: ERK1/2 inhibits Cullin 3/SPOP-mediated PrLZ ubiquitination and degradation to modulate prostate cancer progression

Section 2: Please provide the previous authorship, in the order shown on the manuscript before the changes were introduced. Please indicate the corresponding author by adding (CA) behind the name.

	First name(s)	Family name	ORCID or SCOPUS id, if available
1st author	Yizeng	Fan	
2nd author	Tao	Hou	
3rd author	Yasheng	Zhu	
4th author	Weichao	Dan	
5th author	Bo	Liu	
6th author	Yi	Wei	
7th author	Zixi	Wang	
8th author	Yang	Gao	
9th author	Jin	Zeng	
10th author	Lei	Li (CA)	0000-0003-4928-8722

Please use an additional sheet if there are more than 10 authors.

SPRINGER NATURE Springer Nature is one of the world's leading global research, educational and professional publishers, created in May 2015 through the combination of Nature Publishing Group, Palgrave Macmillan, Macmillan Education and Springer Science+Business Media.

SPRINGER NATURE

Change of authorship request form - Journals (pre-acceptance)

Section 3: Please provide a justification for change. Please use this section to explain your reasons for changing the authorship of your manuscript, e.g. what necessitated the change in authorship? Please refer to the (journal) policy pages for more information about authorship. Please explain why omitted authors were not originally included and/or why authors were removed on the submitted manuscript.

No new author was added.

We changed the authorship order of Weichao Dan and Yasheng Zhu, for that Weichao Dan completed more experiments in the revision stage.

We listed Jin Zeng as one of the co-corresponding author, for that Jin Zeng offered helpful guidance in the revision stage.

Section 4: Proposed new authorship. Please provide your new authorship list in the order you would like it to appear on the manuscript. Please indicate the corresponding author by adding (CA) behind the name. If the Corresponding Author has changed, please indicate the reason under section 3.

	First name(s)	Family name (this name will appear in full on the final publication and will be searchable in various abstract and indexing databases)	Affiliated institute	E-mail address
1st author	Yizeng	Fan	The First Affiliated Hospital of Xi'an Jiaotong University	yzfan1991@163.com
2nd author	Tao	Hou	The First Affiliated Hospital of Xi'an Jiaotong University	houtao1994@126.com
3rd author	Weichao	Dan	The First Affiliated Hospital of Xi'an Jiaotong University	danwc1997@outlook.com
4th author	Yasheng	Zhu	Shanghai Changhai Hospital, Second Military Medical University	574259644@qq.com
5th author	Bo	Liu	The First Affiliated Hospital of Xi'an Jiaotong University	939305754@qq.com
6th author	Yi	Wei	The First Affiliated Hospital of Xi'an Jiaotong University	wairky323@stu.xjtu.edu.cn
7th author	Zixi	Wang	The First Affiliated Hospital of Xi'an Jiaotong University	wzxde1222@163.com
8th author	Yang	Gao	The First Affiliated Hospital of Xi'an Jiaotong University	gaoyangxjtu@126.com
9th author	Jin	Zeng (CA)	The First Affiliated Hospital of Xi'an Jiaotong University	zengjin1984@gmail.com
10th author	Lei	Li (CA)	The First Affiliated Hospital of Xi'an Jiaotong University	lilydr@163.com

Please use an additional sheet if there are more than 10 authors.

SPRINGER NATURE Springer Nature is one of the world's leading global research, educational and professional publishers, created in May 2015 through the combination of Nature Publishing Group, Palgrave Macmillan, Macmillan Education and Springer Science+Business Media.

Change of authorship request form - Journals (pre-acceptance)

Section 5: Author contribution, Acknowledgement and Disclosures. Please use this section to provide a new disclosure statement and, if appropriate, acknowledge any contributors who have been removed as authors and ensure you state what contribution any new authors made (if applicable per the journal or book (series) policy). Please ensure these are updated in your manuscript - after approval of the change(s) - as our production department will not transfer the information in this form to your manuscript.

New acknowledgements:

Not applicable

New Disclosures (financial and non-financial interests, funding):

Conflict of interest
The authors have declared that no competing interest exists.

Funding
This work was supported by grants from the National Natural Science Foundation of China (NO. 81925028, 82073304, 82002694 and 81773206).

New Author Contributions statement (if applicable per the journal policy):

Y.Z.F., T.H., W.C.D. and Y.S.Z. have contributed equally to this work. J.Z. and L.L. designed and supervised all experiments and contributed to the manuscript preparation. Y.Z.F., T.H., W.C.D., Y.S.Z., B.L., Y.W., Z.X.W., Y.G. and J.Z. performed the experiments, analyzed the data and contributed to the manuscript preparation. Y.Z.F., T.H. and Y.S.Z. collected animal samples and participated in the animal experiments. Y.Z.F., J.Z., Y.G. and L.L., wrote the manuscript.

State 'Not applicable' if there are no new authors.

SPRINGER NATURE

Change of authorship request form - Journals (pre-acceptance)

Section 6: Declaration of agreement. *All* authors, unchanged, new and removed *must* sign this declaration.
(NB: Please print the form, (docu)-sign and return/upload a scanned copy. Please note that signatures that have been inserted as an image file are acceptable as long as it is handwritten. Typed names in the signature box are unacceptable.) *** Please delete as appropriate. Delete all of the bold if you were on the original authorship list and are remaining as an author.**

	First name	Family name		Signature	Date
1st author	Yizeng	Fan	I agree to the proposed new authorship shown in section 4 and the proposed change in corresponding author	Yizeng Fan	2022-01-24
2nd author	Tao	Hou	I agree to the proposed new authorship shown in section 4 and the proposed change in corresponding author	Tao Hou	2022-01-24
3rd author	Weichao	Dan	I agree to the proposed new authorship shown in section 4 **/and the addition/removal*of my name to the authorship list** /and the proposed change in corresponding author	Weichao Dan	2022-01-24
4th authors	Yasheng	Zhu	I agree to the proposed new authorship shown in section 4 **/and the addition/removal*of my name to the authorship list** /and the proposed change in corresponding author	Yasheng Zhu	2022-01-24
5th author	BO	Liu	I agree to the proposed new authorship shown in section 4 and the proposed change in corresponding author	Bo Liu	2022-01-24
6th author	Yi	Wei	I agree to the proposed new authorship shown in section 4 and the proposed change in corresponding author	Yi Wei	2022.01.24
7th author	ZiXi	Wang	I agree to the proposed new authorship shown in section 4 and the proposed change in corresponding author	ZiXi Wang	2022.1.24

SPRINGER NATURE Springer Nature is one of the world's leading global research, educational and professional publishers, created in May 2015 through the combination of Nature Publishing Group, Palgrave Macmillan, Macmillan Education and Springer Science+Business Media.

Change of authorship request form - Journals (pre-acceptance)

	First name	Family name		Signature	Date
8th author	Yang	Gao	I agree to the proposed new authorship shown in section 4 and the proposed change in corresponding author	Yang Gao	2022.01.24
9th author	Jin	Zeng	I agree to the proposed new authorship shown in section 4 **/and the addition/removal*of my name to the authorship list** /and the proposed change in corresponding author	Jin Zeng	Jan-23-2022
10th author	LEI	LI	I agree to the proposed new authorship shown in section 4 and the proposed change in corresponding author	[illegible]	Jan-25-2022

Please use an additional sheet if there are more than 10 authors.

In case of author collaborations with formal agreement:

	Name of consortium/consortia	First name	Family name		Signature	Date
Representative/legal guarantor				I agree to the proposed new authorship shown in section 4 **/and the addition/removal*of my name to the authorship list** /and the proposed change in corresponding author		

Both added/removed authors should complete the information in the first table under Section 6.

---- End of form ----

SPRINGER NATURE Springer Nature is one of the world's leading global research, educational and professional publishers, created in May 2015 through the combination of Nature Publishing Group, Palgrave Macmillan, Macmillan Education and Springer Science+Business Media.
